# Serum sodium, tonicity, and risk of dementia in cohort and healthcare populations

**DOI:** 10.1002/alz.71725

**Published:** 2026-08-19

**Authors:** Natalia I. Dmitrieva, Jonathan Rabinowitz, Eric S. Leifer, Delong Liu, Mona N. Bahouth, Manfred Boehm, Rebecca F. Gottesman

**Affiliations:** ^1^ The Laboratory of Cardiovascular Regenerative Medicine National Heart Lung and Blood Institute Bethesda Maryland USA; ^2^ Wellness Lab and Louis and Gabi Weisfeld School of Social Work Bar‐Ilan University Ramat Gan Israel; ^3^ Office of Biostatistics Research National Heart Lung and Blood Institute Bethesda Maryland USA; ^4^ Department of Neurology Johns Hopkins School of Medicine Baltimore Maryland USA; ^5^ Stroke Branch National Institute of Neurological Disorders and Stroke Intramural Research Program Bethesda Maryland USA

**Keywords:** dementia risk, fluid intake, hypertonicity, modifiable risk factors, serum sodium, underhydration

## Abstract

**INTRODUCTION:**

Identifying modifiable, accessible dementia risk factors is critical for prevention. Worldwide surveys indicate that about half of adults do not meet recommended fluid intake and may be chronically underhydrated.

**METHODS:**

We examined associations between intracellular hydration markers and dementia risk in 13,535 participants from the Atherosclerosis Risk in Communities (ARIC) study and 395,802 individuals from Israel's Leumit Health Services. Serum sodium, glucose‐corrected sodium, and tonicity were modeled using restricted cubic splines in adjusted Cox models of incident dementia.

**RESULTS:**

Across both cohorts, with up to 30 years of follow‐up, dementia risk showed a nonlinear association with hydration markers. Risk began increasing within the mid‐normal range, around 141 mmol/L for serum sodium and 287 mosmol/kg for tonicity, reaching a 1.5‐ to 3‐fold increase at the upper end and beyond.

**DISCUSSION:**

These findings suggest hypertonic underhydration as early marker of dementia risk and support adequate water intake and glycemic control for dementia prevention.

## Highlights

- Association of hypertonic underhydration with dementia risk was evaluated
- Serum sodium and tonicity showed nonlinear associations with dementia risk
- Risk increased from mid‐normal sodium (∼141 mmol/L) and tonicity (∼287 mosmol/kg)
- Findings replicated in population and primary care cohorts (up to 30 years of follow‐up)
- Results suggest hypertonic underhydration as a modifiable dementia risk factor.

## 1 BACKGROUND

Dementia is a growing public health concern, affecting more than 57 million individuals worldwide, with cases projected to rise to 153 million by 2050.^1^, ^2^, ^3^. In the United States, annual incident dementia cases among adults aged ≥55 years are projected to increase from approximately 514,000 in 2020 to about 1 million in 2060.^4^. While there is currently no cure, dementia prevention is increasingly viewed as a feasible public health strategy. According to the 2020 and 2024 Lancet Commission, up to 40% of dementia cases could be prevented or delayed by addressing known modifiable risk factors across the life course.^1^, ^5^, ^6^. Nonetheless, the global burden of dementia remains high, especially in settings where current prevention strategies may be difficult to implement.^1^. There is a critical need to identify additional, *easily modifiable and globally accessible* risk factors to expand prevention potential.

Hydration status—specifically hypertonic underhydration (intracellular dehydration driven by elevated effective osmolality)—has recently emerged as a promising, understudied contributor to chronic disease risk.^7^. Growing evidence from population‐based cohorts and experimental studies indicates that markers of hypertonic underhydration—elevated serum sodium, serum tonicity, and vasopressin (AVP) levels—are associated with increased risks of hypertension^8^, diabetes^9^, heart failure^8^, ^10^, ^11^, kidney disease^12^, accelerated aging and premature mortality.^13^, ^14^ Notably, these risks begin to rise even at mild, subclinical levels of hypertonic underhydration, when biomarkers remain within current normal reference ranges.^7^ In free‐living populations, inadequate water intake is a common, modifiable contributor to hypertonic underhydration.^15^, ^16^, ^17^ Because many people consume less water than recommended,^18^, ^19^ promoting adequate water intake, while addressing other contributors such as increased water loss, reduced thirst sensitivity, or diminished renal concentrating ability^20^, ^21^, ^22^ may be a low‐cost, broadly implementable public‐health strategy to reduce chronic disease burden and preserve cognitive health. This hypothesis is supported by evidence from mouse model of lifelong water restriction, which show reduced lifespan and accelerated age‐related degenerative changes.^23^

Hydration status may also be directly relevant to brain aging. The brain is highly sensitive to changes in extracellular tonicity because osmotic gradients alter cellular water movement, requiring neuronal and glial osmotic adaptation to preserve cell volume and function.^24^ Experimental studies of mild hypohydration have shown short‐term impairments in attention, working memory, mood, perceived task difficulty, and stress level, indicating that even modest water deficit can affect brain function.^25^, ^26^, ^27^ AVP, which is stimulated by elevated tonicity, may further connect hypertonic underhydration to dementia risk through vascular, metabolic, renal, and stress‐related pathways.^28^, ^29^, ^30^ However, direct prospective evidence linking midlife intracellular hydration biomarkers within the normal clinical range to incident dementia remains limited, representing an important research gap.

In this study, we examined the association between midlife hypertonic underhydration and the long‐term risk of dementia. We assessed intracellular hydration using serum biomarkers‐serum sodium, glucose‐corrected sodium, and calculated tonicity as exposure variables. These measures capture distinct physiological aspects of water balance regulation: sodium is the primary contributor to extracellular osmolality^20^; glucose‐corrected sodium accounts for hyperglycemia‐induced dilutional hyponatremia, thereby improving classification of intracellular hydration status^31^, ^32^; and tonicity (effective osmolality)^33^ reflects the osmotic pressure sensed by osmoreceptor neurons which stimulate AVP release and initiate water‐conservation responses in states of hypertonic underhydration.^7^, ^34^, ^35^, ^36^, ^37^ Because AVP secretion is tightly regulated by changes in tonicity, measures such as glucose‐corrected sodium and calculated tonicity may provide a more physiologically relevant assessment of hypertonic underhydration than unadjusted sodium alone, by incorporating the osmotic effects of glucose.

**RESEARCH IN CONTEXT**

1. **Systematic review**: We reviewed the literature on hydration, cognitive performance, and dementia. Prior studies report associations between dehydration and poorer cognitive and physical performance, as well as a high prevalence of dehydration among individuals with dementia. However, we found no longitudinal studies evaluating subclinical underhydration as a risk factor for incident dementia.
2. **Interpretation**: Our findings show increased risk of dementia among individuals with serum sodium > 141 mmol/L or tonicity > 287 mosmol/kg‐levels within the normal range that may reflect activation of water‐conservation mechanisms. These results suggest subclinical hypertonic underhydration may be an early, modifiable risk factor for dementia.
3. **Future directions**: Future research should include (1) interventional trials to test whether improving hydration reduces dementia risk and whether serum sodium and tonicity can guide targeted interventions, and (2) studies on the long‐term effects of hydration on brain structure and function. Promoting adequate hydration within existing intake recommendations may represent a low‐risk, immediately actionable strategy to support brain health.

We first conducted analyses in the Atherosclerosis Risk in Communities (ARIC) Study, a well‐characterized U.S. population‐based cohort with long‐term follow‐up and carefully adjudicated incident dementia. To evaluate generalizability and potential clinical implementation, we then replicated key associations using routine healthcare data from 395,802 individuals in Israel's Leumit Health Services system. Our objective was to investigate whether intracellular hydration‐related biomarkers, serum sodium and tonicity, within the normal clinical range are associated with dementia risk, and whether these associations hold in both controlled cohort data and real‐world healthcare settings.

## 2 METHODS

### 2.1 Dataset

We used data from the ARIC study (https://www5.cscc.unc.edu/aric9/about/project_overview). The main datasets were obtained from the NHLBI Biologic Specimen and Data Repository Information Coordinating Center (BioLINCC). Apolipoprotein E4 (ApoE4) allele genomic data were obtained from ARIC study investigators. The datasets were redacted to remove personal identifiers to conform to the individual informed consent restrictions. Transfer of datasets was approved by the National Institutes of Health (NIH) Office of Human Subjects Research (OHSR) and was excluded from Institutional Review Board review as Not Human Subjects Research, based on the interpretation of 45 CRF 46 under “Research Involving Coded Private Information or Biological Specimens” and Guidance on Engagement of Institutions in Human Subjects Research (October 16, 2008).

### 2.2 Study population

ARIC is an ongoing population‐based prospective cohort study in which 15,792 45‐ to 64‐year‐old mostly Black and White men and women were enrolled from four US communities (Foryth County, NC; Jackson, MS [Black individuals only]; Washington County, MD; and Minneapolis suburbs, MN) and seen at a baseline visit in 1987–1989. A detailed original study design description has been published.^38^ Each ARIC community cohort was selected by probability sampling to ensure that all individuals in the eligible age group had equal chances of being selected. After the baseline visit, there were three subsequent in‐person visits at approximately three‐year intervals (visit 2 in 1990–1992; visit 3 in 1993–1995; visit 4 in 1996–1998), followed by visit 5 in 2011–2013, which constituted the first ARIC Neurocognitive study (ARIC‐NCS) visit, with subsequent ongoing regular visits that also incorporated ARIC‐NCS assessments. Participants have been contacted annually (and subsequently, semiannually starting around 2012) since baseline, to obtain additional information about hospitalizations and for additional data collection. For the current analysis, data accumulated by the end of 2020 were used, which also coincided with the end of visit 8. For the purposes of this analysis, the study baseline is defined as visit 2, as we defined our hydration markers based on the average of values from visits 1 and 2. Detailed overview of the ARIC study timeline is provided at the study website (https://www5.cscc.unc.edu/aric9/about/project_overview). The ARIC Study protocol was approved by the institutional review board of each participating center and informed consent was obtained from participants or their proxies at each study visit.

### 2.3 Exclusions

**FIGURE 1:**
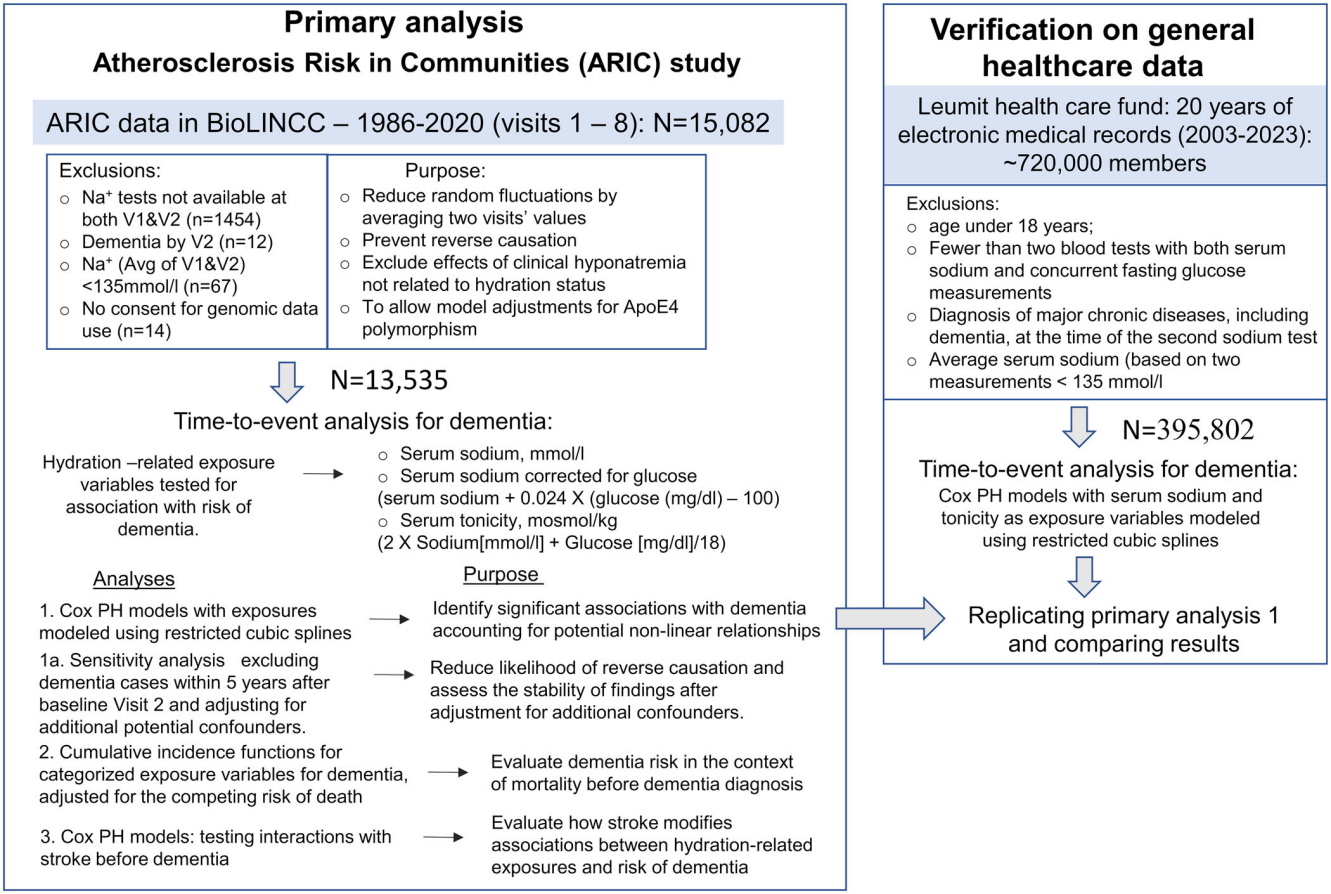
Outline of the study datasets and analyses.

Out of 15,792 participants originally enrolled in ARIC who completed the visit 1 examination, a total of 15,082 participants were available in the data obtained through BioLINCC after standard ARIC exclusions were completed (such as for exclusion of the very small numbers of individuals self‐reporting as being from a race other than Black or white; or being of Black race in Washington County, MD or Minneapolis, MN), and also excluded individuals who did not consent for the data sharing. We subsequently excluded 1454 individuals who did not attend the visit 2 examination which was used as baseline for the current study analyses. Twelve individuals were excluded for whom surveillance identified an onset of dementia prior to visit 2. We further excluded 67 participants who had an average sodium concentration from visits 1 and 2 lower than 135 mmol/L, to avoid inclusion of clinical conditions and medications that consistently result in hyponatremia.^39^ Out of the remaining 13,549 participants, 14 did not give consent to use genomic data that were needed for ApoE4 status and were also excluded, leaving 13,535 individuals for primary analysis (Figure 1).

### 2.4 Exposure variables

Three exposure variables representing measures of the study participants baseline intracellular hydration status were evaluated: serum sodium, serum sodium corrected for glucose, and serum tonicity.

#### 2.4.1 Blood collection and laboratory measurements

At ARIC study visits 1 (1987–1989) and 2 (1990–1992), venous blood samples were collected from participants who were asked to fast overnight. Blood was drawn via standard venipuncture and processed to separate serum, which was then stored under controlled conditions for laboratory analysis. Serum sodium concentrations were measured using an indirect ion‐selective electrode method, and serum glucose concentrations were determined using the hexokinase enzymatic assay. All measurements were performed in central laboratories following standardized protocols and quality control procedures established by the ARIC study to ensure accuracy and consistency across visits. Detailed descriptions of laboratory methods and quality assurance procedures are available in the ARIC cohort manuals (https://aric.cscc.unc.edu/aric9/researchers/manuals).

#### 2.4.2 Serum sodium

This was calculated as the average serum sodium at visits 1 and 2, which occurred approximately 3 years apart. Averaging was performed to minimize random fluctuations of test results or test day hydration, since our subsequent analysis was conducted under the assumption that the average of these two serum sodium measurements more accurately reflects an individual's hydration habits.^40^, ^41^

#### 2.4.3 Serum sodium corrected for glucose

The sodium corrected for hyperglycemia ^31^, ^32^ was calculated from the average sodium and average glucose concentrations at visits 1 and 2. We employed the Hillier method, which provides a more accurate assessment of intracellular hydration status by adjusting sodium for the osmotic effects of glucose. The correction was performed using the following formula: Corrected Sodium = Serum Sodium + 0.024 × (Glucose [mg/dl] ‐ 100). This is equivalent to adding 1 mmol/L to the measured sodium level per 42 mg/dl increase in glucose above 100 mg/dl as shown in Table S1.^32^ This adjustment helps to mitigate the dilutional effect of hyperglycemia on sodium concentration. Elevated glucose draws water from the intracellular to the extracellular space due to its hypertonic properties, leading to a reduction in serum sodium despite the presence of intracellular dehydration. Correcting sodium for glucose addresses this discrepancy by estimating the serum sodium concentration that would produce equivalent cellular dehydration effects, thereby providing a more accurate measure of the individual's true intracellular hydration status in the setting of hyperglycemia.

#### 2.4.4 Serum tonicity

This was calculated from serum sodium and glucose levels using the formula: Tonicity [mOsmol/kg] = 2 × Sodium [mmol/L] + Glucose [mg/dl] / 18.^33^ By reflecting only effective osmoles, tonicity is the key trigger for arginine‐vasopressin‐mediated water conservation ^35^ and thus provides a physiologically grounded index of cellular hydration. Serum sodium represents 95–98% of the serum tonicity at normal glucose levels. However, with increasing glucose levels, tonicity provides a more accurate estimation of intracellular hydration status, similar to serum sodium correction for glucose.

For our analyses, the exposure variables were analyzed as either continuous variables modeled as restricted cubic splines or categorized into five equal intervals within the 135–150 mmol/L range observed in the study cohort for sodium and glucose‐corrected sodium (five groups: 135–137.5; 138–140.5; 141–143.5; 144–146.5; 147–150 mmol/L) and within the 270–310 mosmol/kg range for tonicity (five groups: ≥270– < 278; ≥278– < 286; ≥286– < 294; ≥294– < 302; ≥302– < 310) mosmol/L).

### 2.5 Outcomes

Incident dementia over nearly 30 years of follow‐up (from visit 2 [1990–1993] through 2020) was the primary outcome of interest. Cognitive assessment was limited to a brief in‐person neuropsychological battery at ARIC visits 2 and 4, with a more extensive neuropsychological battery and informant interviews for cognitive status at ARIC‐NCS visits (visits 5–8+). A dementia diagnosis was made based on precise algorithms developed for participants undergoing in‐person assessment or phone‐based cognitive assessment, for telephone informant interviews and for diagnosis based on hospital discharge diagnostic codes, with expert (physician and neuropsychologist) review and confirmation of these algorithmic diagnoses. Detailed description of the ascertainment methodology for cognitive impairment and dementia is described in prior ARIC Neurocognitive Study publications.^42^ Follow‐up time for those with incident dementia was the time from the visit 2 exam (i.e., the baseline time for our analysis) until the incident event. For cumulative incidence functions (CIF) analyses, death without dementia was included as a competing risk.

### 2.6 Covariates

Several covariates were used for adjustments of statistical models and for exclusions. Sex and race were self‐identified at visit 1. Education level, also self‐reported at visit 1, was divided in three categories: less than completed high school; completed high school or vocational school; or at least some college or higher degree. Smoking status at visit 2 was self‐reported. Genotyping of the *APOE* ε4 allele, predisposing to dementia, was completed using TaqMan system (Applied Biosystems, Foster City, CA, USA). Presence of *APOE* ε4 allele was defined as being heterozygous or homozygous for the allele. Blood pressure (BP) was measured three times after 5 minutes of rest and recorded as average of the two last measurements, and high BP was defined as systolic BP > 140 mm Hg or diastolic BP > 90 mm Hg; hypertension at visit 2 was defined as high (BP) as measured during the visit 2 examination or taking BP medications (participants brought in current medications which were reviewed).

Other variables that were used in the study analyses were plasma glucose (mg/dl, average of visits 1 and 2), low‐density lipoprotein (LDL) cholesterol level (mg/dl, visit 2), body mass index (BMI) (kg/m^2^, visit 2), and diabetes (visit 2), defined as fasting glucose > 140 mg/dl, or taking diabetes medications). Stroke, used in some sensitivity analyses, and modeled as a time‐varying covariate, was defined as an expert‐adjudicated ischemic or hemorrhagic stroke with date of onset before dementia.

Baseline physical activity, alcohol intake, salt intake, and BP medications were used in sensitivity analysis that assessed robustness of the findings to potential confounding. In ARIC, physical activity was assessed using an interviewer‐administered questionnaire adapted from the Baecke physical activity questionnaire, which captured usual physical activity over the prior year across work, sport/exercise, and leisure‐time domains. For model adjustment, we used the derived sport physical activity index, scored on a scale from 1 to 5 in 0.25‐point increments, with higher values indicating greater activity; details of the assessment and scoring methods have been described previously.^43^ Alcohol consumption was assessed using an ARIC‐derived variable based on self‐reported usual weekly intake of wine, beer, and liquor. Total ethanol intake was calculated in grams per week using beverage‐specific ethanol weights, with former and never drinkers assigned a value of 0 g/week. Salt intake habits were assessed using an interviewer‐administered questionnaire and categorized as typically adding salt to meals at the table versus not adding salt. BP medication use, including diuretic use, was evaluated in stratified analyses to assess whether medication use may have influenced the observed associations.

### 2.7 Statistical analyses

An overview of the analyses conducted in this study is presented in Figure 1. Descriptive statistics were used to summarize baseline characteristics of the analytic cohort and compare demographic and clinical variables across exposure categories. We employed Cox proportional hazards (PH) time‐to‐event models to examine the respective associations, in separate models, between each of three hydration‐related exposure variables described above (serum sodium, serum sodium corrected for glucose, and tonicity) and the risk of incident dementia. Each exposure variable was modeled using restricted cubic splines to account for potential non‐linear relationships. To evaluate the added predictive value of each hydration‐related variable, likelihood ratio tests were used to compare multivariable Cox PH models with and without, respectively, serum sodium, glucose‐corrected sodium, or tonicity.

All models were adjusted for age, sex, race, education level, presence of the ApoE4 allele, hypertension status, smoking status, LDL cholesterol, and BMI. Covariates were used from visit 2, which served as the study baseline. Results of the time‐to‐event analyses are reported as hazard ratios (HRs) with corresponding 95% confidence intervals (CIs).

We also evaluated potential effect modification by testing interactions between each hydration‐related exposure variable and selected demographic, genetic, and clinical variables. Specifically, separate interaction models were fitted for serum sodium, glucose‐corrected sodium, and tonicity with race, sex, ApoE4 status, hypertension, diabetes, blood pressure‐lowering medication use, renal function assessed by estimated glomerular filtration rate (eGFR), and stroke before dementia. Hydration‐related exposure variables were modeled as restricted cubic splines in these analyses. For each interaction test, we compared a reduced Cox model containing the main effects with a full model additionally including the interaction between the spline‐modeled hydration‐related variable and the selected covariate. Overall interaction *p*‐values were obtained using likelihood ratio tests comparing the reduced and full models. Interactions were further evaluated visually using stratified hazard ratio spline plots.

To further characterize the rate of dementia onset over the follow‐up period in relation to baseline exposure levels, each exposure variable was divided into five equal‐range intervals (see the exposures section). For each group, we generated CIFs accounting for death as a competing risk, as well as overall survival probability curves. Statistical analyses were conducted with R (version 4.4.1, *R Core Team*, 2024) executed within RStudio (version 2024.04.2; Posit PBC, Boston, MA, USA) and SAS® 9.4 for Windows (SAS Institute Inc., Cary, NC, USA; Copyright © 2023). Graphs were produced using GraphPad Prism 9.5.1 for Windows (GraphPad Software, Boston, MA, USA; www.graphpad.com).

### 2.8 Sensitivity analyses

We conducted several sensitivity analyses to evaluate the robustness of the findings. First, to reduce the likelihood of reverse causation, we performed a lagged analysis excluding 15 participants who developed dementia within 5 years after baseline, defined as visit 2, as well as 759 participants who were lost to follow‐up or censored during this period leaving 12,761 individuals in the sensitivity analyses cohort. In this analysis, follow‐up began 5 years after visit 2. This approach was used because dementia diagnosed shortly after visit 2 may have been developing before or during the exposure assessment period.

In a second sensitivity analysis, using the original visit 2 baseline, we included stroke as a time‐dependent covariate in Cox models. Likelihood ratio tests were used to evaluate whether hydration‐related predictors were independently associated with incident dementia after accounting for stroke that occurred before dementia.

We also performed additional sensitivity analyses to assess potential confounding by factors related to hydration status or dementia risk. These models were additionally adjusted for baseline salt intake habits, kidney function, alcohol intake and physical activity. Salt intake habits were assessed at baseline by classifying participants according to whether they typically added salt to meals at the table. Kidney function was assessed using eGFR, modeled as a continuous variable, and chronic kidney disease (CKD) was defined as eGFR < 60 ml/min/1.73 m^2^. Because impaired kidney function may influence sodium and water balance, we also repeated analyses after excluding participants with CKD.

### 2.9 Leumit healthcare medical records

To assess the generalizability of findings from the ARIC cohort and their potential relevance for routine clinical settings, we conducted a replication analysis using electronic medical records from the Leumit Health Services, one of Israel's four national healthcare providers (https://www.innovation.leumit.co.il/about). Leumit maintains a centralized database with over 20 years of nationwide medical data from approximately 700,000 individuals. For the current study, we included 395,802 adults aged 18–105 years who had at least two serum sodium and fasting glucose measurements and no prior diagnosis of dementia or major chronic diseases at the time of their second sodium test, which served as the baseline for analysis. Individuals with serum sodium < 135 mmol/L were excluded to avoid including subjects with clinically significant water balance disturbances.^39^ Detailed information on cohort construction and exclusions has been published previously.^8^

Serum sodium and calculated serum tonicity were used as exposure variables and modeled as restricted cubic splines to allow for non‐linear associations, following the same approach as for the ARIC analyses. The primary outcome was incident dementia, identified using International Classification of Diseases – Revision 9 (ICD‐9) diagnostic codes in the medical record after the baseline visit. Time‐to‐event analyses were performed using Cox PH models adjusted for age and sex. No additional covariates were included due to incomplete availability of other clinical variables at the baseline time point.

## 3 RESULTS

### 3.1 Study population

The primary time‐to‐event analysis for dementia risk was performed on the 13,535 ARIC study participants who remained after exclusions (see Figure 1 and methods for details). The characteristics of the participants for the whole cohort and for the categorical groups by exposure category are presented in Table 1, Table S2 and S3. The average age at visit 2, which was the baseline for these analyses, was 57 years. During nearly 30 years of follow‐up, 2965 (22%) participants developed dementia. The cohort was 55% female, 24% Black individuals, 31% carried at least one ApoE4 allele; 23% were smokers at the time of visit 2; and 30% had hypertension. The participants had an average BMI of 28 kg/m^2^, and LDL cholesterol of 133 mg/dl. Age was generally similar across sodium level categories, but individuals with higher sodium levels were more frequently women, more frequently of Black race and had lower education level.

**TABLE 1:** Demographic and clinical characteristics of ARIC participants by serum sodium groups

		Serum sodium groups, mmol/L (average of V1&V2)				
Baseline characteristics	All participants (*N* = 13535)	135–137.5 (*N* = 699)	138–140.5 (*N* = 5508)	141–143.5 (*N* = 6450)	144–146.5 (*N* = 857)	147–150 (*N* = 21)
Age, years – mean (SD)	57.1 (5.7)	56.4(5.9)	56.7(5.8)	57.3(5.6)	57.9(5.5)	56.9(5.0)
Serum Na^+^, mmol/L – mean (SD)						
Average of V1&2	140.9 (1.9)	136.9(0.7)	139.6(0.8)	142.0(0.8)	144.6(0.7)	147.9(0.9)
Visit 1	141.0 (2.4)	137.0(1.7)	139.6(1.5)	142.1(1.5)	144.8(1.7)	148.6(3.2)
Visit 2	140.8 (2.3)	136.8(1.7)	139.6(1.5)	141.9(1.5)	144.3(1.7)	147.2(2.9)
Glucose, mg/dl: Avg V1&2	111.0(38.1)	145.4(79.5)	112.8(41.7)	106.7(26.0)	103.5(17.1)	106.2(28.4)
Sex – no. (%)						
Female	7400 (55)	387 (55)	2812 (51)	3622 (56)	562 (66)	17 (81)
Male	6135 (45)	312 (45)	2696 (49)	2828 (44)	295 (34)	4 (19)
Race – no. (%)						
White	10301 (76)	550 (79)	4407 (80)	4807 (75)	532 (62)	5 (24)
Black	3234 (24)	149 (21)	1101 (20)	1643 (25)	325 (38)	16 (76)
Current smoker – no. (%)						
Yes	3043 (23)	197 (28)	1256 (23)	1385 (22)	200 (24)	5 (24)
No	10457 (77)	500 (72)	4243 (77)	5048 (78)	650 (76)	16 (76)
Hypertension^*^ – no. (%)	4854 (36)	283 (41)	1895 (34)	2308 (36)	357 (42)	11 (52)
ApoE4 – no. (%)	4030 (31)	185 (28)	1588 (30)	1979 (32)	274 (33)	4 (20)
BMI^†^ – mean (SD)	28.0 (5.4)	28.4 (5.7)	27.8 (5.2)	28.0 (5.4)	28.5 (5.9)	30.9 (9.1)
LDL Chol^#^ – mean (SD)	134 (37)	128 (36)	131 (37)	136 (37)	139 (37)	135 (29)
Education – no. (%)						
< High school	2936 (22)	130 (19)	1126 (20)	1437 (22)	236 (28)	7 (33)
HS or GED	5631 (42)	302 (43)	2253 (41)	2730 (43)	341 (40)	(24)
> HS	4945 (37)	267 (38)	2124 (39)	2267 (35)	278 (32)	9 (43)
Diabetes – no. (%)	1597 (12)	224 (32)	715 (13)	599 (9)	57 (7)	2 (10)
Incident dementia						
No. (%)	2965 (22.1)	124 (17.8)	1145 (20.9)	1471 (23.0)	218 (25.7)	7 (33)
Rate per 1000 person‐years	10.4	9.1	9.8	10.7	12.6	17.1

*Note*: Baseline characteristics at visit 2 for participants included in the time‐to‐event analysis for dementia (*N* = 13,535). Participants were grouped by average serum sodium concentration from visits 1 and 2 into five equal‐width intervals (135‐150 mmol/L). Shown are demographic and clinical variables, including mean sodium concentrations at each visit, average glucose levels, and the dementia incidence rates observed during follow‐up within each group. See Tables S2 and S3 for equivalent analyses based on glucose‐corrected serum sodium and calculated serum tonicity.

Abbreviations: ApoE4, apolipoprotein E4; ARIC, Atherosclerosis Risk in Communities; GED, general education development; HS, high school;

* Hypertension is defined as systolic blood pressure > 140 mmHg or diastolic blood pressure > 90 mmHg, or use of antihypertensive medications.

^†^ BMI: body mass index, calculated as weight (kg) divided by height (m^2^).

^#^ LDL Chol: low‐density lipoprotein cholesterol, in mg/dl.

The analyses in this study were based on the assumption that serum sodium remains relatively stable over time and reflects habitual hydration status for each individual. This assumption is supported by prior analyses of clinical records from two independent health system US cohorts (Veterans Integrated Service Networks: 143,973 participants, 688,611 sodium determinations; Kaiser NW: 30,216 participants, 89,973 determinations), serum sodium exhibited substantial within‐person stability over a 10‐year period ^40^. In our prior ARIC analysis, two measurements 3 years apart correlated positively (*r* = 0.36; 95% CI: 0.34–0.37; *p* < 0.0001), and between‐individual variance exceeded within‐individual variance (5.62 vs. 1.78 (mmol/L)^2^), indicating nonrandom, person‐level stability over time.^41^ In the present analysis, average serum sodium concentrations at visits 1 and 2 were nearly identical within each sodium‐defined group, further confirming the stable distribution of this measure over time (Table 1).

Notably, participants in the lowest sodium group had the highest average serum glucose levels and highest proportion of diabetes (Table 1), a pattern consistent with the dilutional effect of hyperglycemia.^31^ Elevated glucose concentrations increase extracellular tonicity, drawing water out of cells and leading to a relative dilution of extracellular sodium levels despite cellular dehydration. Correction of serum sodium for glucose (Sodium + 0.024 × (Glucose [mg/dl]‐100)^32^) (Tables S1 and S2) and calculation of serum tonicity (2 × Sodium [mmol/L] + Glucose [mg/dl] / 18) ^33^ (Table S3) reduced average glucose concentrations and proportion of diabetes in the lower exposure groups, indicating that these adjusted measures more accurately reflect true intracellular hydration status by accounting for the osmotic effects of glucose.

Crude dementia rates were higher in the higher serum sodium groups, and were nearly twice as frequent in the highest sodium group (147–150; 17.1 per 1000 person‐years), albeit with a smaller sample size (*n* = 21), than in the lowest sodium group (135–137.5; 9.1 per 1000 person‐years).

### 3.2 Serum sodium, tonicity, and risk of dementia: Time‐to‐event analysis

**FIGURE 2:**
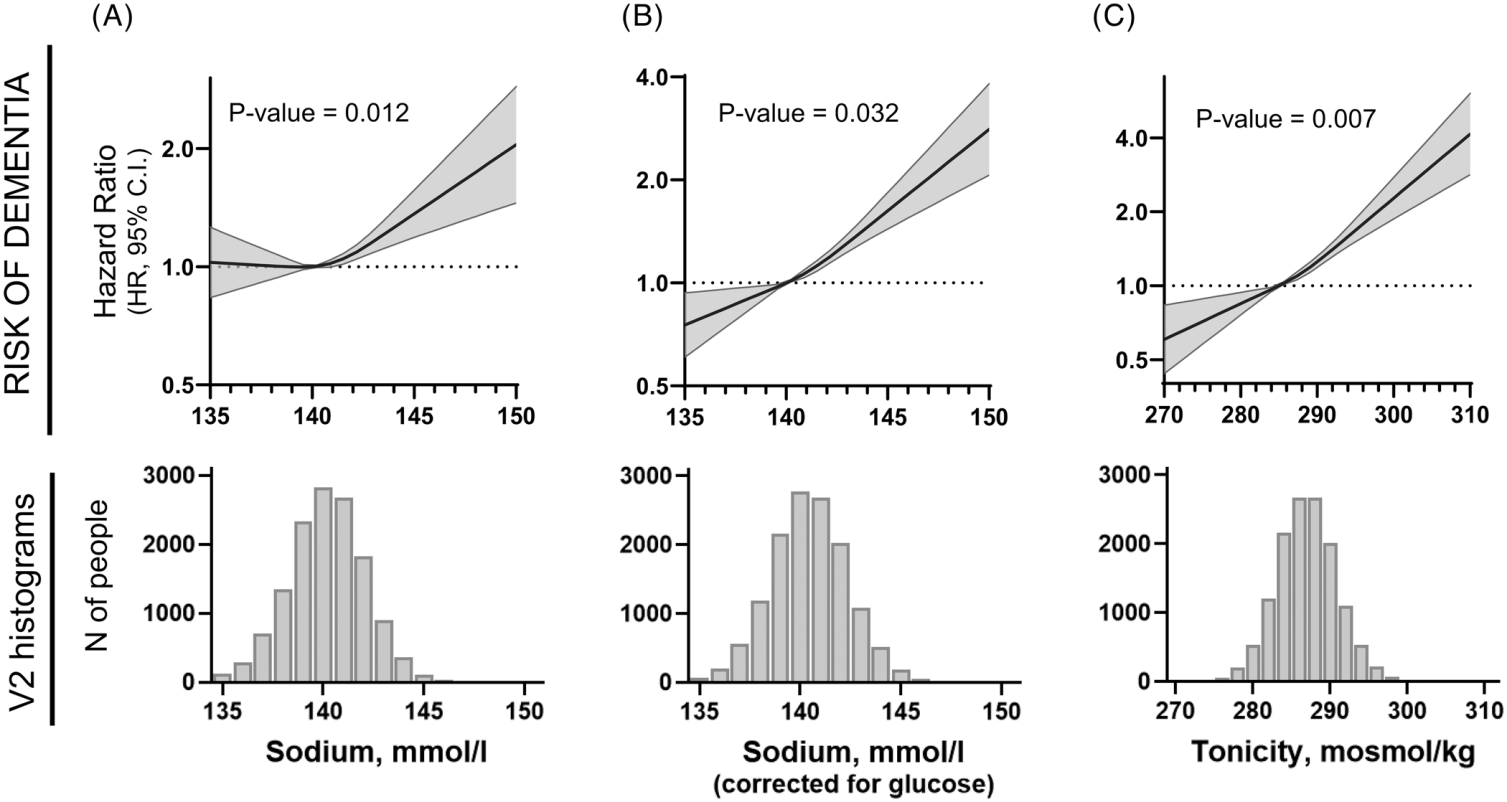
Evaluation of the association between hydration‐related biomarkers and dementia risk using Cox proportional hazards (PH) models. (A–C) The three hydration‐related predictor variables: (A) serum sodium, (B) glucose‐corrected serum sodium, and (C) serum tonicity. Upper panels: Hazard ratios (HRs) for incident dementia are shown as a function of each predictor variable, modeled using restricted cubic splines in unadjusted Cox PH models. Splines are plotted relative to 140 mmol/L for serum sodium and 285 mOsm/kg for serum tonicity. Shaded areas indicate 95% confidence intervals (CIs). *p*‐Values shown on each panel represent results from likelihood ratio tests (LRTs), which compare the fit of fully adjusted Cox models with and without the corresponding hydration‐related predictor variable to assess its added predictive value. Adjustment covariates include age, sex, education, apolipoprotein E4 (ApoE4) status, smoking status, hypertension, low density lipoprotein (LDL) cholesterol, and body mass index (BMI) at visit 2. All three hydration markers significantly improved model fit. See Figure S2 for the first derivative plots used to identify threshold points at which dementia risk begins to accelerate. Lower panels: Histograms showing the distribution of baseline values for each hydration‐related biomarker in the study population.

As an initial step in examining the association between baseline hydration‐related variables and the risk of developing dementia, we conducted time‐to‐event analyses using Cox PH models. Serum sodium, glucose‐corrected sodium, and tonicity were modeled as restricted cubic splines in separate models to allow for non‐linear relationships. In multivariable‐adjusted models—including covariates for age, sex, education, presence of the ApoE4 allele, smoking status, hypertension, LDL cholesterol, and BMI—the likelihood ratio tests indicated that all three exposure variables significantly improved model fit (Figure 2). Plots of the HR curves revealed non‐linear associations between each exposure and dementia risk, showing increasing risks starting in the upper half of normal ranges (Figure 2) with inflection points suggesting increased risk at serum sodium and glucose‐corrected sodium concentrations above 140‐141 mmol/L, and at tonicity values exceeding 287‐289 mosmol/kg (Figure S1).

### 3.3 Dementia risk in the context of mortality: Competing risk and survival analyses of hydration‐related exposure variables

Given previously reported associations between higher serum sodium levels and increased mortality in the ARIC cohort^13^, we performed additional analyses to account for death as a competing risk when evaluating dementia risk. We used CIF to estimate dementia incidence while accounting for pre‐dementia death as a competing risk and compared these patterns with Kaplan–Meier survival curves.^44^

**FIGURE 3:**
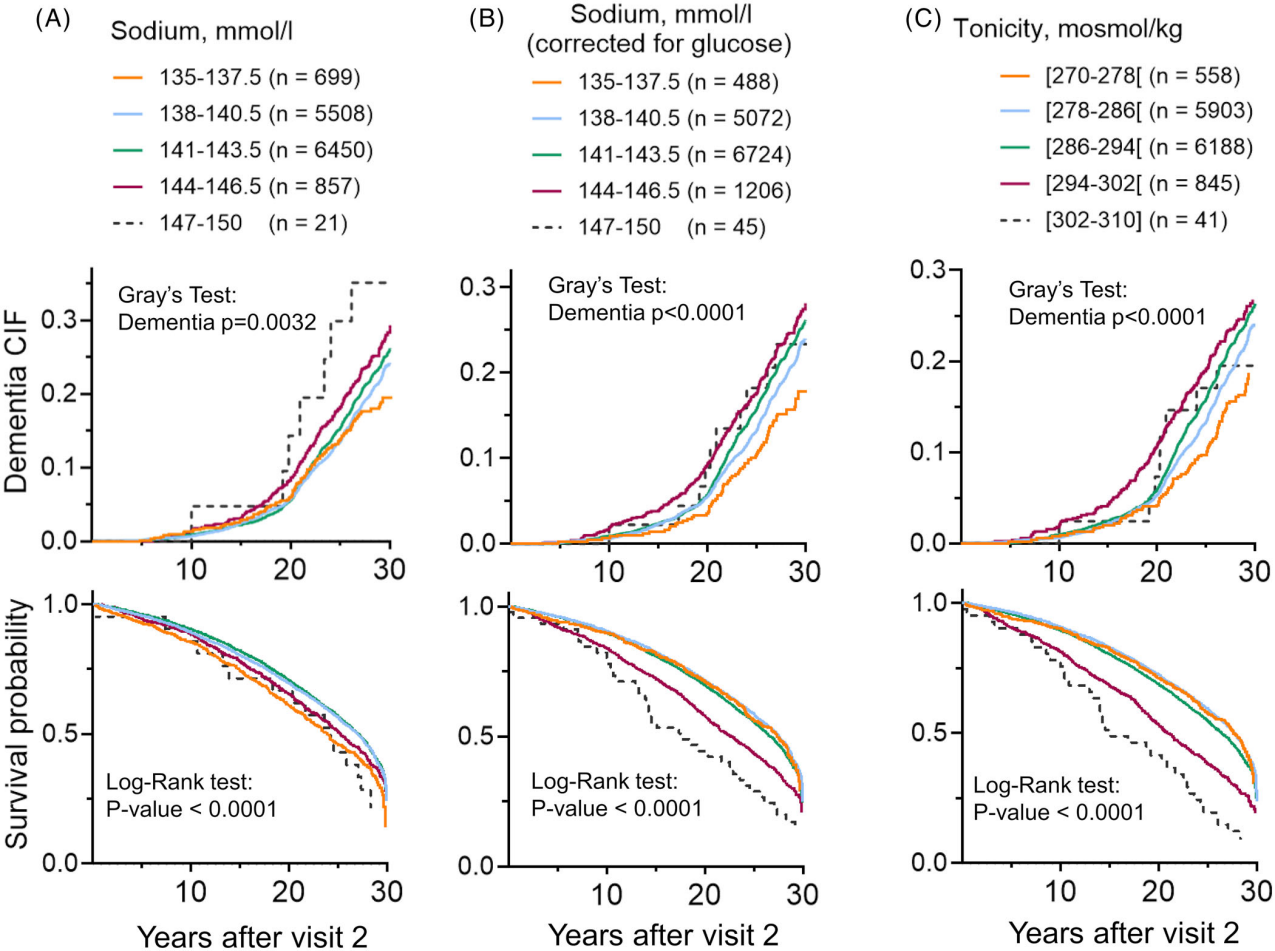
Cumulative dementia incidence and overall survival by hydration‐related predictor groups. Hydration‐related variables were evaluated as follows: (A) serum sodium, (B) glucose‐corrected serum sodium, and (C) serum tonicity. For each variable, the observed range was divided into five equal‐width intervals, as shown in the figure panels. Upper panels: Cumulative incidence functions (CIFs) for dementia are plotted over time, accounting for the competing risk of death. Hydration‐level group differences in dementia risk were statistically tested using Gray's test for equality of CIFs. The significant *p*‐values indicate differences in the CIFs across the hydration groups for all three exposure variables. Lower panels: Kaplan–Meier survival curves for all‐cause death across the hydration‐level groups. Statistical comparisons were performed using the log‐rank test. The significant *p*‐values indicate differences in survival across the hydration groups for all three exposure variables.

Figure 3 provides the CIFs for dementia across categories of hydration‐related exposure variables, along with corresponding Kaplan–Meier survival curves. The CIFs (upper panels) demonstrate significant differences in dementia incidence across all exposure groups, with Gray's test yielding *p*‐values < 0.001. Higher categories of sodium and tonicity are associated with higher cumulative incidence of dementia. The Kaplan–Meier survival curves (lower panels) confirm previously reported associations between higher sodium‐related measures and reduced overall survival, with the log‐rank tests showing significant differences between the groups (*p* < 0.001).

After glucose correction the lowest serum sodium group (135–137.5 mmol/L) decreased from 699 (Figure 3A) to 488 participants (Figure 3B) because individuals with hyperglycemia were reclassified into higher glucose‐corrected sodium groups. This reclassified lowest glucose‐corrected sodium group had the lowest observed cumulative incidence of dementia (Figure 3B). This reclassification highlights the utility of glucose correction for more accurately capturing intracellular hydration status by estimating the sodium concentration that reflects equivalent cellular dehydration effects. Similarly, the lowest tonicity group had the lowest cumulative incidence of dementia (Figure 3C).

Consistent with the spline‐based analyses (Figure 2), dementia incidence increased progressively across higher exposure groups for all three variables, with a steeper rise and earlier separation in the 144–146.5 mmol/L sodium group; 144–146.5 mmol/L sodium group also had higher mortality than the lower exposure groups, indicating that higher dementia incidence in this group occurred despite shorter vital follow‐up. Elevated mortality in the highest exposure groups was also evident for glucose‐corrected sodium and tonicity (Figures 3B–C).

### 3.4 Sensitivity analysis

To assess the robustness of the findings, we conducted sensitivity analyses, including a lagged analysis to address reverse causation (Figure S2). In the lagged analysis, 15 participants who developed dementia within the first 5 years after visit 2 and 759 participants who were lost to follow‐up or censored during this period, were excluded. Results were similar to the primary analyses, supporting the robustness of the associations (Figure S2).

Additional sensitivity analyses considered additional adjustment for potential confounders, including salt intake, kidney function (eGFR and CKD), alcohol intake and physical activity. Because high salt intake, particularly in settings of impaired kidney function, could contribute to higher serum sodium, we evaluated these potential influences in additional analyses. In baseline comparisons, participants in the lower serum sodium and tonicity groups were more likely to report adding salt at the table than those in the higher groups, suggesting that higher salt intake was unlikely to be the primary driver of elevated serum sodium in this cohort (Table S4). Mean eGFR did not differ meaningfully across serum sodium groups (Table S5); however, the higher sodium groups included a greater proportion of participants with CKD (defined as eGFR < 60 ml/min/1.73 m^2^) (Table S5).

**FIGURE 4:**
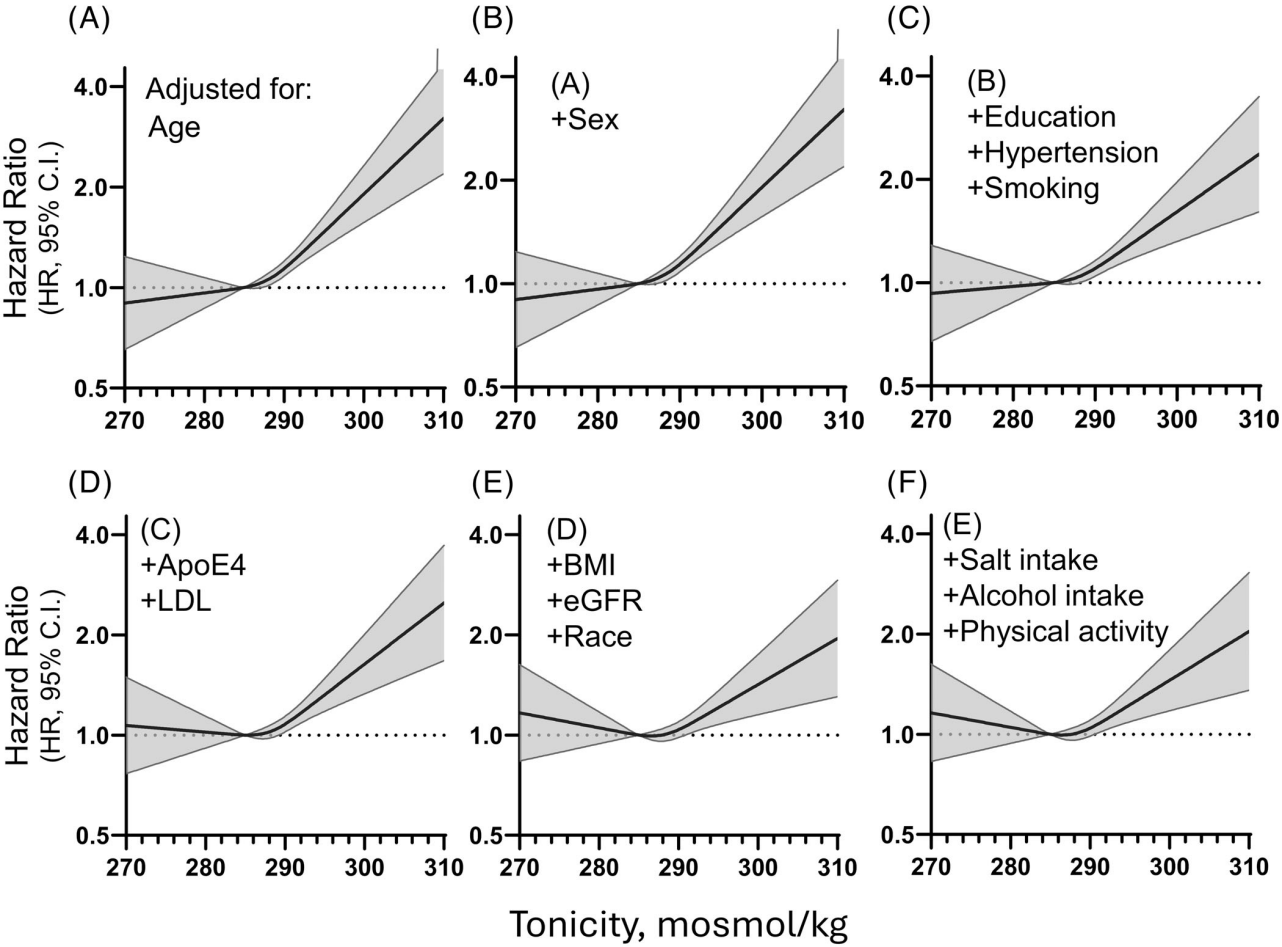
Sensitivity analysis: stability of the tonicity‐dementia association across sequentially adjusted Cox models. Hazard ratios (HRs) for incident dementia were estimated using Cox PH models with serum tonicity modeled as a restricted cubic spline and referenced to 285 mOsm/kg. Shaded areas represent 95% confidence intervals (CIs). Models were sequentially adjusted to evaluate whether the spline association was attenuated or changed in shape after progressive adjustment for potential confounders: (A) age; (B) age and sex; (C) plus education, hypertension, and smoking status; (D) plus apolipoprotein E4 (ApoE4) status and low‐density lipoprotein (LDL) cholesterol; (E) plus body mass index (BMI), estimated glomerular filtration rate (eGFR), and race; and (F) plus salt intake, alcohol intake, and physical activity.

In Cox models, likelihood ratio tests showed that all hydration‐related exposure variables remained significant predictors of dementia risk after additional adjustment for salt intake, eGFR, alcohol intake and physical activity, and after excluding 233 individuals with eGFR < 60, ml/min/1.73 m^2^ (Table S6). Among the three hydration‐related exposures, tonicity showed the strongest model improvement, consistent with the interpretation that glucose‐corrected measures better characterize intracellular hydration status than serum sodium alone. In sequentially adjusted models based on tonicity, the spline curves showed a consistent increase in dementia risk at higher tonicity levels, with only slight attenuation or change in shape after progressive adjustment for demographic, lifestyle and clinical covariates (Figure 4).

### 3.5 Stroke and risk of dementia: Interaction with hydration‐related exposure variables

Because stroke is an established risk factor for dementia, including in the ARIC study,^45^, ^46^ we evaluated the associations between hydration‐related variables and dementia risk after accounting for incident stroke during follow‐up and tested whether these associations differed by stroke status. An overview of the analyses is shown on Figure S3.

**FIGURE 5:**
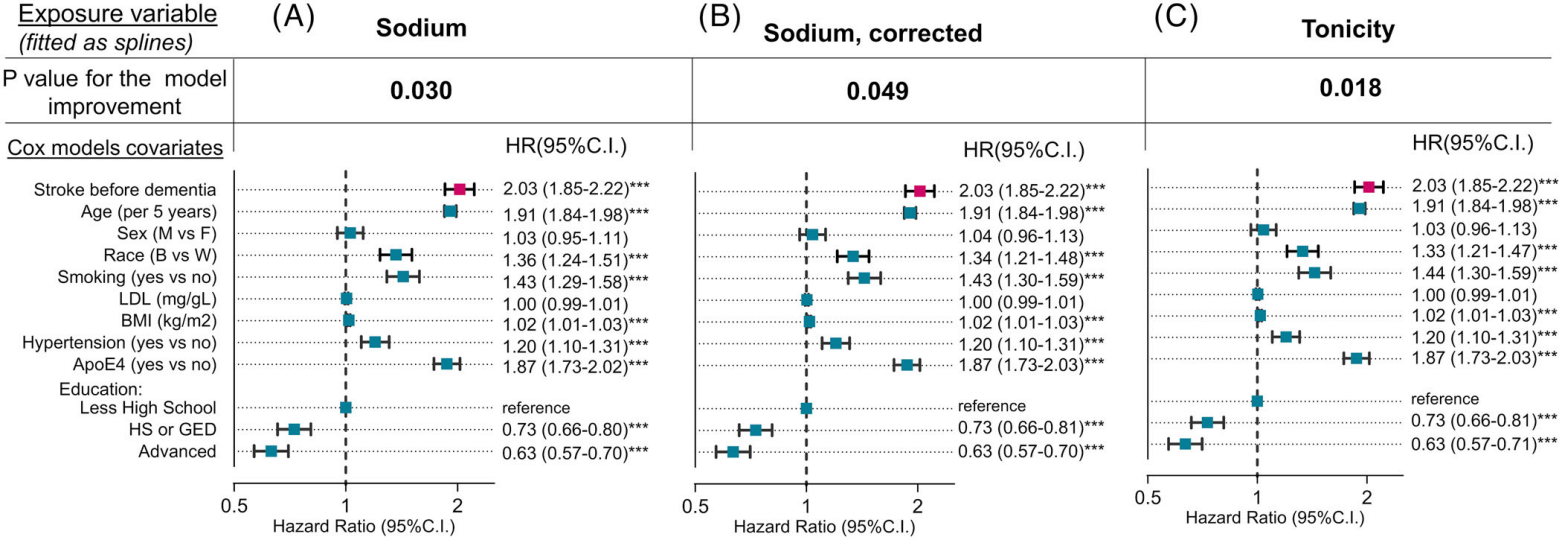
Evaluation of the independent effects of hydration‐related biomarkers on dementia risk after adjusting for stroke as a time‐dependent covariate. This analysis tests whether serum sodium, glucose‐corrected sodium, and serum tonicity remain significant predictors of dementia after accounting for stroke events that occur prior to dementia diagnosis. Stroke was included as a time‐dependent variable in the Cox proportional hazards (PH) models: (A) serum sodium, (B) glucose‐corrected serum sodium, and (C) serum tonicity. Each hydration‐related variable was modeled as a restricted cubic spline and added to the multivariable Cox model used in the main analysis (Figure 1), now additionally adjusted for stroke. *p*‐Values from likelihood ratio tests (LRTs), shown on each panel, compare model fit with and without the corresponding hydration‐related variable. Covariates in all models include age, sex, education, apolipoprotein E4 (ApoE4) genotype, smoking status, hypertension, low density lipoprotein (LDL) cholesterol, and BMI measured at visit 2. Results are presented as forest plots, displaying hazard ratios (HRs) and 95% confidence intervals (CIs) for all covariates in each model. All three hydration‐related variables remained statistically significant contributors to model fit, indicating that their associations with dementia risk are independent of stroke occurrence.

After inclusion of stroke events occurring during follow‐up before dementia diagnosis as a time‐varying covariate in adjusted Cox PH models, serum sodium, glucose‐corrected sodium, and tonicity remained associated with dementia risk when modeled as restricted cubic splines. (*p*‐values: 0.018‐0.049) (Figure 5 and S3B).

**FIGURE 6:**
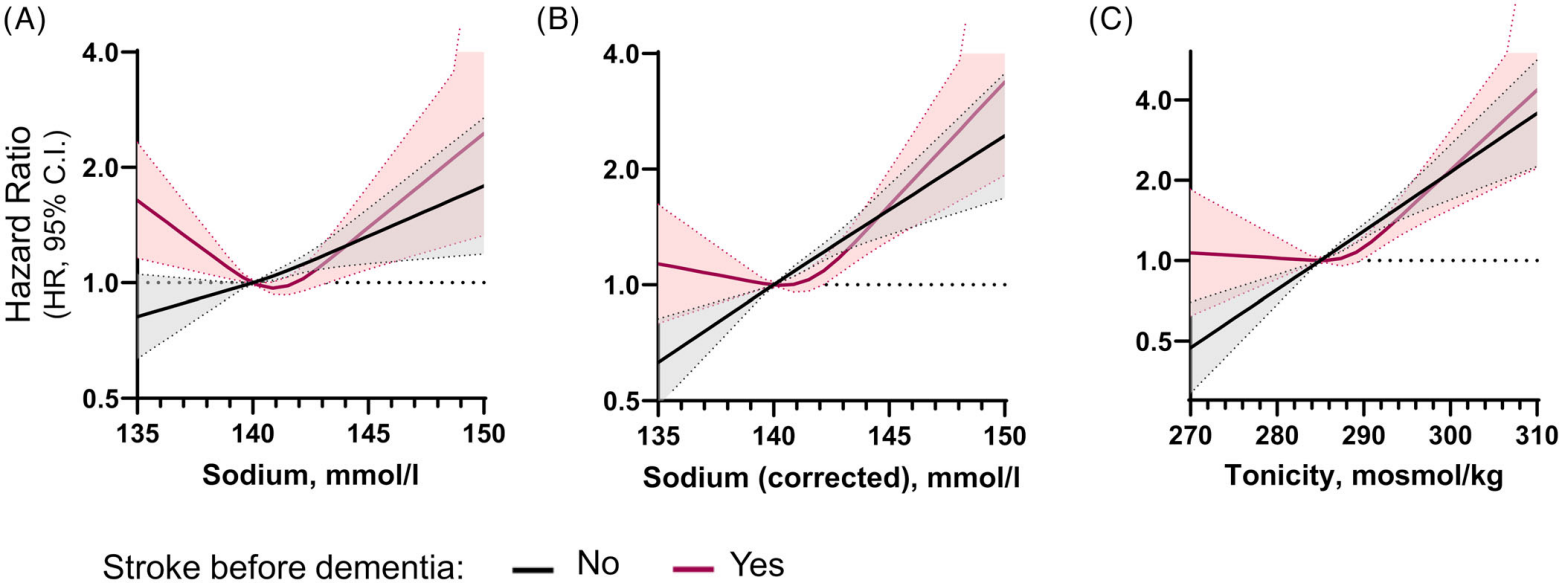
Stratified analysis of dementia hazard ratio (HR) splines by stroke before dementia diagnosis. Spline‐based HR curves for incident dementia are shown separately for participants who experienced stroke prior to dementia diagnosis and those who did not. Splines are plotted relative to 140 mmol/L for serum sodium and 285 mOsm/kg for serum tonicity. Panels represent different hydration‐related predictors: (A) serum sodium, (B) glucose‐corrected sodium, and (C) serum tonicity. At the higher end of the exposure range, HRs gradually increased for both stroke strata.

To assess potential effect modification by stroke, we also tested interaction terms between time‐varying stroke and each hydration‐related variable (Figure S3C). A statistically significant interaction was observed for serum sodium (Table S7). To explore this interaction, we stratified participants into two groups: those who experienced a stroke before dementia diagnosis (stroke group) and those who did not (no‐stroke group). Within each group, HR curves for serum sodium, glucose‐corrected sodium, and tonicity were re‐estimated using spline models (Figure 6). At the lower end of the serum sodium range, the stroke group showed increased dementia risk, a pattern not observed in the no‐stroke group. This elevated risk was attenuated after correcting sodium for glucose and was not observed when tonicity was used as the exposure, suggesting that the apparent effect is driven by misclassification of hydration status due to hyperglycemia—similar to the pattern observed in the CIF analysis (Figure 3).

At the higher end of the exposure range, HRs gradually increased for both stroke strata, beginning around the same thresholds observed in the overall spline models (approximately 140‐141 mmol/L for sodium and 287‐289 mosmol/kg for tonicity) (Figure 6).

We detected no significant interactions between intracellular hydration markers and sex, race, or ApoE4 status with respect to dementia incidence (Table S7). We also tested interaction with selected clinical variables, including diabetes, hypertension, and renal function assessed by eGFR; none showed significant interactions (Table S7), Stratified spline analyses by baseline diabetes status (Figure S4), hypertension status (Figure S5A), antihypertensive medication use including diuretics (Figure S5B), and salt intake habits (Figure S6) showed similar patterns of increasing dementia risk at higher levels of hydration‐related variables across strata. In diabetes‐stratified Cox models, individuals with diabetes showed increased dementia risk at the lower end of uncorrected serum sodium; this pattern was attenuated in models using glucose‐corrected sodium and tonicity (Figure S4).

### 3.6 Using serum sodium and glucose as joint predictors of dementia risk

Our preceding analyses indicate that evaluating serum sodium alone, without accounting for glycemic status, may lead to misclassification of intracellular hydration status. Therefore, correcting sodium for glucose or using calculated serum tonicity as an exposure variable provides a more accurate assessment of dementia risk. To translate these findings into a practical risk stratification framework, we examined the joint distribution of measured serum sodium and glucose in relation to predicted dementia risk using spline‐based HR estimates from the tonicity model.

**FIGURE 7:**
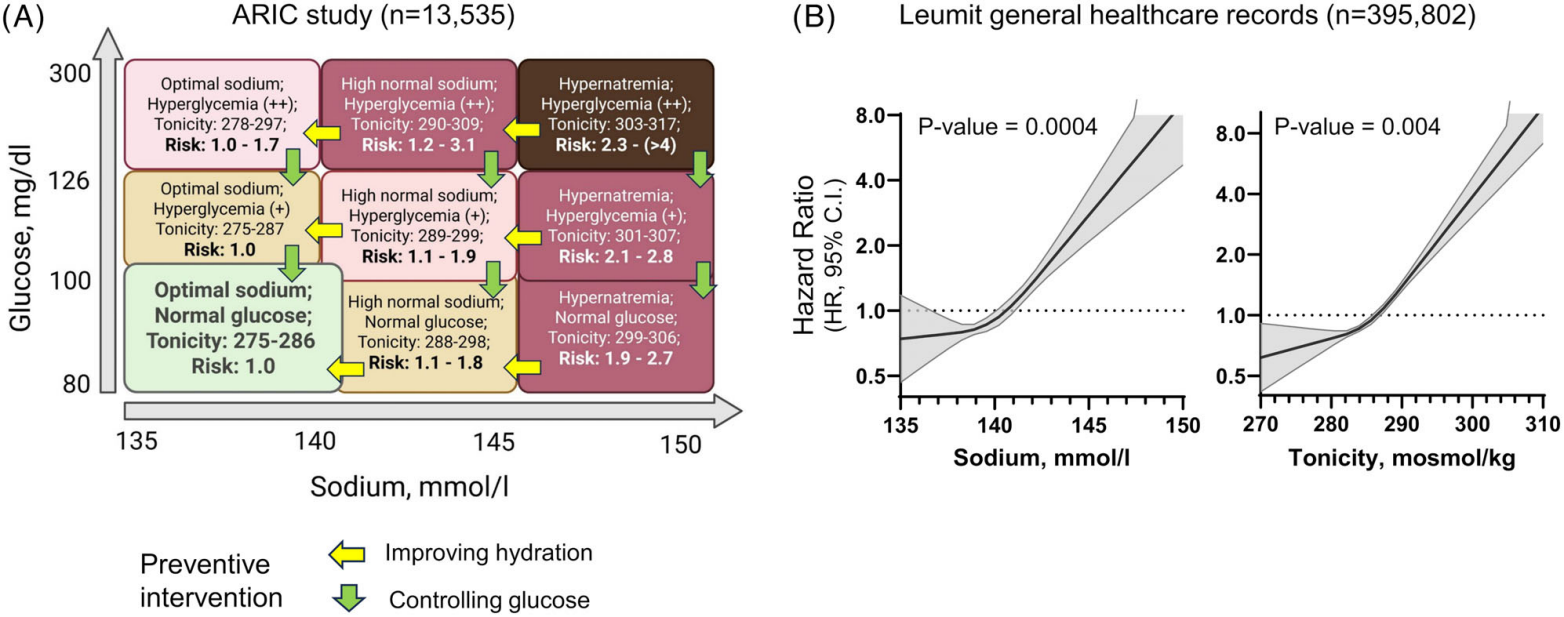
Risk stratification and external validation of hydration‐related biomarkers for dementia prediction. (A) Predicted dementia risk by combined serum sodium and glucose status in the Atherosclerosis Risk in Communities (ARIC) cohort. Participants were classified into nine groups based on serum sodium (categorized as optimal, high‐normal, or hypernatremia) and glucose status (normal, mild hyperglycemia [+], and moderate to severe hyperglycemia [++]). For each group, ranges of serum tonicity and predicted dementia risk were estimated using spline‐based hazard ratios from the ARIC tonicity model. The highest predicted dementia risks were observed among individuals with both elevated sodium and severe hyperglycemia, highlighting a potential strategy for early risk identification and prevention through improved hydration and glycemic control. Source data for this panel are provided in Tables S8 and S9. (B) External validation of spline‐based associations in a separate dataset of 395,802 individuals from routine healthcare records from Israel's Leumit Health Services. Unadjusted Cox models showed consistent hazard ratio (HR) curves for sodium and tonicity, with similar shapes and risk thresholds as observed in the ARIC cohort. In models adjusted for age and sex, *p*‐values from likelihood ratio tests confirmed significant improvement in model fit with the inclusion of hydration‐related exposures, supporting the generalizability and clinical relevance of these biomarkers for dementia risk prediction across populations.

In Figure 7A, participants were classified into nine groups based on measured serum sodium, categorized as optimal, high‐normal, and hypernatremia, and glucose status, categorized asnormal, mild hyperglycemia, and moderate to severe hyperglycemia. For each group, we calculated the corresponding ranges of serum tonicity (Table S8) and used the tonicity model to estimate dementia risk. HRs were calculated relative to a reference interval of 270–286 mosmol/kg (Table S9). Predicted dementia risk was higher in groups with higher serum sodium, higher glucose, and corresponding higher calculated tonicity. The highest risks were observed among individuals with both elevated sodium and hyperglycemia.

### 3.7 External validation in a routine healthcare data

To validate the generalizability of these findings, we conducted an independent analysis using healthcare records from approximately 395,802 individuals in a general healthcare setting. Figure 7B shows the HR curves for serum sodium and tonicity, modeled as restricted cubic splines in Cox proportional hazard models. The shape of the HR curves and the threshold values for increased dementia risk closely mirror those observed in the ARIC cohort, with increased risk beginning around 140–141 mmol/L for sodium and 287–289 mosmol/kg for tonicity. Likelihood ratio tests showed significant model improvement when sodium and tonicity were included to Cox models adjusted for age and sex.

## 4 DISCUSSION

### 4.1 Principal findings

In this study, we investigated the relationship between midlife intracellular hydration status—measured by serum sodium, glucose‐corrected sodium, and serum tonicity—and the long‐term risk of developing dementia. Using data from a well‐characterized, prospective U.S. cohort (ARIC), we identified non‐linear associations between these hydration‐related markers and incident dementia. Risk began to rise at serum sodium levels above 140‐141 mmol/L and tonicity above 287‐289 mosmol/kg, despite values remaining within the clinical normal range (Figure 2). These associations persisted after accounting for death as a competing risk (Figure 3) and after adjustment for multiple potential confounders (Figure 4), supporting the robustness of the findings. Importantly, higher dementia incidence in the highest sodium and tonicity groups was observed despite higher mortality and shorter vital follow‐up, suggesting that the association was not explained by differential survival.

Glucose correction appeared to improve classification of intracellular hydration status, particularly among individuals with hyperglycemia or diabetes, in whom uncorrected serum sodium may underestimate the osmotic burden. Consistently, models using glucose‐corrected sodium or tonicity attenuated the increased risk observed at the lower end of uncorrected sodium, suggesting that tonicity may better capture the combined contribution of sodium and glucose to cellular dehydration. Associations also remained after accounting for incident stroke, and higher sodium and tonicity were associated with increased dementia risk across stroke strata (Figure 6), suggesting that these relationships are not fully explained by clinically recognized stroke.

In addition to the robust associations found, with consistent relationships across sensitivity analyses, these results were validated in 395,802 individuals from Leumit Health Services, with similar hazard ratio shapes and risk thresholds (Figure 7B). Together, these findings suggest that mild, chronic hypertonic underhydration, reflected in routinely measured laboratory values, is associated with higher risk of dementia, and may help identify individuals who could benefit from preventive intervention targeting hydration status and glycemic control (Figure 7A).

### 4.2 Biological plausibility and relationship to prior evidence

This work builds on a growing body of research linking intracellular hydration status to long‐term health outcomes. In mouse model, lifelong water restriction, accelerated age‐related degenerative changes and shortened lifespan.^23^ Prior epidemiologic and mechanistic studies have shown that subtle elevations in serum sodium and tonicity are associated with increased risk of cardiovascular and metabolic diseases, faster biological aging, and premature mortality.^7^ The current study extends these findings to dementia risk.

Notably, the risk thresholds we observe correspond to sodium and tonicity levels that trigger secretion of AVP (vasopressin),^36^, ^37^ linking our observational findings to biologically plausible mechanisms of water balance regulation. Our use of glucose‐corrected sodium and tonicity as refined indicators of intracellular hydration further strengthens the physiological relevance of these metrics because they account for osmotic shifts associated with hyperglycemia.^31^, ^32^

### 4.3 Serum sodium and tonicity as markers of long‐term hydration status

A central premise of our approach is that serum sodium reflects an individual's long‐term hydration habits. This is supported by previous clinical record analyses showing that sodium concentrations remain stable within individuals over a 10‐year period, clustering around person‐specific “set points”.^40^ Our prior work in ARIC, excluding hyperglycemia cases, showed that sodium values were stable within 2–3 mmol/L between visits 1 and 2.^41^ These findings suggest that midlife sodium levels likely represent durable individual traits although longer repeated‐measure studies in diverse populations are needed.

Our analyses also indicated that the association of serum sodium and tonicity with dementia risk were independent of salt intake in this cohort, suggesting that higher salt intake was unlikely to be the primary driver of elevated serum sodium. Among individuals with intact kidney function, serum sodium is only minimally influenced by habitual salt intake because of the kidney's high capacity to excrete sodium. Intervention trials report very small differences in serum sodium across sustained salt intake ranges typical of global populations (e.g., ∼0.4 mmol/L between 5–6 g/day vs 10–12 g/day).^47^, ^48^ In contrast, population differences in fluid intake are large ranging from approximately 0.7 to 3–4 L/day ^18^, ^19^, and based on the Adrogué‐Madias formulation, 1 L of electrolyte‐free water is expected to lower serum sodium in an 80‐kg adult by approximately 3 mmol/L.^20^ Thus, real‐world differences in fluid intake likely have a substantially larger impact on serum sodium than differences in salt intake.

### 4.4 Strengths and limitations

This study has multiple strengths. The use of flexible spline modeling enabled us to capture non‐linear associations without imposing restrictive assumptions. Models were carefully adjusted for key demographic, genetic, and clinical variables. By including stroke as a time‐varying covariate, we accounted for its dynamic role as a potential mediator. Additional strengths include stroke‐adjusted and stratified analyses, development of a sodium‐glucose risk stratification chart, and external validation in a large clinical dataset.

Limitations include the observational design, which prevents causal inference. Despite adjustment for relevant confounders, residual confounding is possible. Selection bias may also have been introduced by excluding participants with missing data, particularly those who did not attend visit 2. Because nonattendance at visit 2 may have been related to poorer health, the analytic cohort may have been healthier than the original ARIC population.

Medication use may also have influenced hydration‐related biomarkers. Although we accounted for hypertension that included blood pressure medications, including identified diuretic users, other medications that may affect fluid balance, sodium levels, glucose levels, or renal handling were not fully captured. Hydration and glucose status were evaluated at only two time points, which may not fully capture within‐person variability over time, and fasting before blood draws may have influenced hydration status. Although sodium correction for glucose and use of tonicity enhance physiological accuracy, these remain indirect measures, that primarily reflect intracellular hydration and do not capture extracellular volume status, which is needed for complete assessment of hydration. Stroke subtype and severity were not included, and the Leumit dataset lacked complete covariate information, limiting model adjustment. Despite these caveats, the consistency of findings across cohorts and analytic methods reinforces the robustness of the observed associations.

### 4.5 Clinical translation and public health implications

Our findings address two major challenges in dementia prevention: early identification and implementation feasibility. Intracellular hydration markers measured in midlife were associated with long‐term risk, highlighting a potential window for early intervention. Because sodium and glucose are routinely measured in clinical care, these markers could be incorporated into existing infrastructure and risk assessment workflows without specialized testing. The consistency between the ARIC and Leumit supports the generalizability of these markers across populations and real‐world healthcare systems.

Clinical translation should emphasize risk identification and individualized evaluation rather than a universal recommendation to increase water intake. As previously proposed in the context of heart failure prevention ^49^, elevated serum sodium should prompt assessment of potential causes, including low habitual fluid intake, increased water losses, impaired renal concentrating ability, medication use, or other medical conditions. For generally healthy individuals with low fluid intake, improving hydration toward recommended intake levels may represent a low‐cost and scalable preventive strategy. However, individuals with CKD, heart failure, hyponatremia risk, diuretic use, or other conditions affecting electrolyte and water balance may require individualized clinical guidance before changing fluid intake. Thus, the real‐world application of these findings is a feasible screening and prevention framework in which routinely measured sodium and glucose identify individuals who may benefit from further hydration assessment, appropriate optimization of fluid intake, and improved glycemic control.

These findings may also help expand the current dementia prevention framework. Established early and midlife risk factors, including low education, hypertension, diabetes, and obesity, have combined population attributable fractions (PAFs) suggesting that up to 40% of dementia cases may be preventable.^1^, ^5^ Hydration status—captured via routinely available markers—has not previously been considered in these models. Because hypertonic underhydration‐as indicated by elevated sodium and tonicity‐arises primarily when water intake is insufficient to compensate for ongoing water loses,^20^ adequate water intake to match daily losses may represent a globally accessible, low‐cost, and scalable prevention target (Figure 7A, Supplementary Graphical abstract). Worldwide surveys suggest that roughly half of adults do not meet recommended fluid intake and may be chronically underhydrated.^18^, ^19^ This points to a broad opportunity for hydration‐focused prevention within existing recommendations for adequate water intake, while also recognizing that causality and optimal intervention approaches require further study.

### 4.6 Future directions and conclusion

Future studies should explore causality through interventional trials, evaluate repeated hydration measures over longer time periods, and assess outcomes in more diverse populations. In parallel, risk prediction models and clinical guidelines may benefit from incorporating serum sodium and tonicity as early markers of dementia vulnerability that may be modifiable through adequate water intake and glycemic control.

In conclusion, midlife hydration‐related biomarkers within the clinical reference range were associated with long‐term dementia risk in ARIC and were externally validated in a large routine healthcare dataset. These findings suggest that mild chronic hypertonic underhydration may be an underrecognized and potentially modifiable contributor to dementia risk. Because serum sodium and glucose are routinely measured, they may offer a practical approach for identifying individuals who could benefit from hydration assessment, individualized guidance, and future prevention strategies.

## CONFLICT OF INTEREST STATEMENT

The authors have no financial conflicts of interest. Author disclosures are available in the Supporting Information.

## DISCLOSURES

Author disclosures are available in the supporting information.

## CONSENT STATEMENT

The Atherosclerosis Risk in Communities (ARIC) study was approved by institutional review boards at all participating centers, and all participants provided written informed consent. Data were obtained through the NHLBI Biologic Specimen and Data Repository Information Coordinating Center (BIOLINCC). The Leumit Health Services study was approved by the Leumit Health Services institutional review board. The present analyses used de‐identified data, and did not require additional informed consent.

## Supporting information



Supporting Information
