# Supplementary material for: Serum sodium, tonicity, and risk of dementia in cohort and healthcare populations
Source: Alzheimers Dement. 2026 Aug 19;22(8):e71725. doi: 10.1002/alz.71725 (PMC13490916; doi:10.1002/alz.71725)
Supplement: Supplementary file 1 [file ALZ-22-e71725-s001.pdf]

Supplement

Serum sodium, tonicity, and risk of dementia in cohort and healthcare populations

Table of contents

Graphical Abstract………………………………………………………………………………… .2

Supplemental Figures:

Figure S1 (related to Figure 2). Threshold identification for accelerated dementia risk associated with serum sodium (A), glucose-corrected sodium (B) and tonicity (C)... 3

Figure S2 (related to Figure 2). Sensitivity analysis for the association between hydration-related biomarkers and dementia risk, excluding early dementia cases and short follow-up...............................4

Figure S3. Modeling stroke's role in associations between hydration-related biomarkers and dementia risk. Conceptual diagram of the analyses shown on Figures 5, 6, Table S6...5

Figure S4 (related to Table S6). Stratified analysis of dementia hazard ratio (HR) splines by baseline diabetes status...6

Figure S5 (related to Table S6). Stratified analysis of dementia hazard ratio (HR) splines by baseline hypertension status and antihypertensive medication use …………… 7

Figure S6 (related to Table S6). Stratified analysis of dementia hazard ratio (HR) splines by baseline salt intake habits…………………………………………….. ……………. 8

Supplemental Tables:

Table S1. Algorithm used to create Sodium corrected for glucose variable...9

Table S2 (related to Table 1). Demographic and clinical characteristics of ARIC participants by serum glucose-corrected sodium group...10

Table S3 (related to Table 1). Demographic and clinical characteristics of ARIC participants by serum tonicity groups..11

Table S4. Assessment of salt intake in relation to serum sodium and tonicity among ARIC participants based on the habit of adding salt at the table to already prepared food.
…………………………………………………………………………………………………12

Table S5. Assessment of baseline kidney function in relation to serum sodium and tonicity among ARIC participants based on eGFR measured at Visit 2. ……………………………………………………13

Table S6. Sensitivity analysis of model improvement after additional covariate adjustment………..…14

Table S7 (related to Figure 6). Interaction analysis between hydration-related biomarkers and demographic/clinical variables...15

Table S8 (related to Figure 7A). Calculated serum tonicity values corresponding to different combinations of sodium and glucose concentrations, used for dementia risk estimation in conjunction with Table S8..16

Table S9 (related to Fig. 7A). Hazard ratios for incident dementia across serum tonicity values relative to the 270–286 mosmol/kg reference range...17

Graphical Abstract

Background: The number of people living with dementia is rapidly increasing due to rising longevity. Preventive interventions targeting modifiable and accessible risk factors are needed to prolong dementia-free lifespans. Worldwide surveys indicate that about half of adults do not meet recommended fluid intake and may be chronically underhydrated..

Key question: Are hypertonic underhydration markers - serum sodium and tonicity - associated with an increased risk of dementia?

Key finding: Two populations were analyzed: 13,535 individuals enrolled in a 30-year cohort study in four US communities and 395,802 individuals from 20 years of medical records within Israel's general healthcare services. Analyses identified increasing risk for developing dementia starting in the middle of normal ranges: around 141 mmol/L for serum sodium and 287 mosmol/kg for tonicity (2 x Sodium + Glucose/18). These levels represent approximate thresholds for activation of water conservation mechanisms through triggering secretion of antidiuretic hormone in response to increased extracellular tonicity.

Take-home message: Hypertonic underhydration is a modifiable dementia risk factor with serum tonicity exceeding 287 mosmol/kg associated with increased risk. Preventive intervention may include ensuring adequate water intake - aiming for serum sodium below 141 mmol/l - and glycemic control.

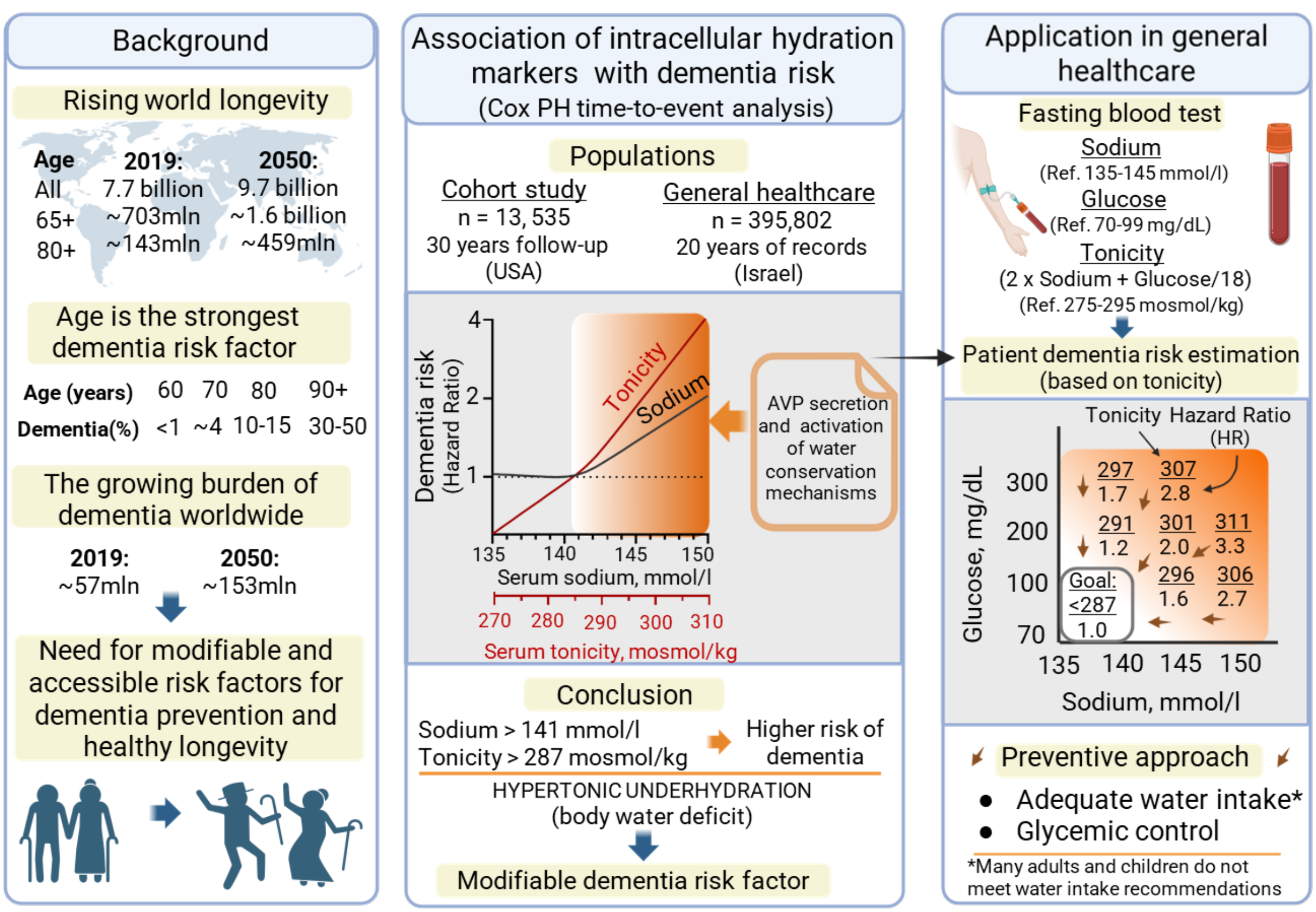

Supplemental Figures

Figure S1 (related to Figure 2). Threshold identification for accelerated dementia risk associated with serum sodium (A), glucose-corrected sodium (B) and tonicity (C).
Upper panels: Hazard ratios (HRs) for dementia as a function of serum sodium, glucose-corrected sodium, and tonicity, modeled as restricted cubic splines from unadjusted Cox proportional hazards models (as in Figure 2).
Lower panels: First derivatives of the HR spline functions are plotted to identify inflection points where the rate of dementia risk increase accelerates. Arrows mark the threshold concentrations where this acceleration begins—approximately 139–140 mmol/L for both serum sodium and glucose-corrected sodium, and 285–287 mosmol/kg for tonicity. These inflection points fall within the middle of the clinical normal ranges and correspond to physiological levels that trigger AVP (arginine vasopressin) release, suggesting a potential link between subclinical underhydration and elevated dementia risk. While these associations are consistent with activation of water conservation pathways, the observational nature of the analysis limits causal interpretation and warrants further investigation.

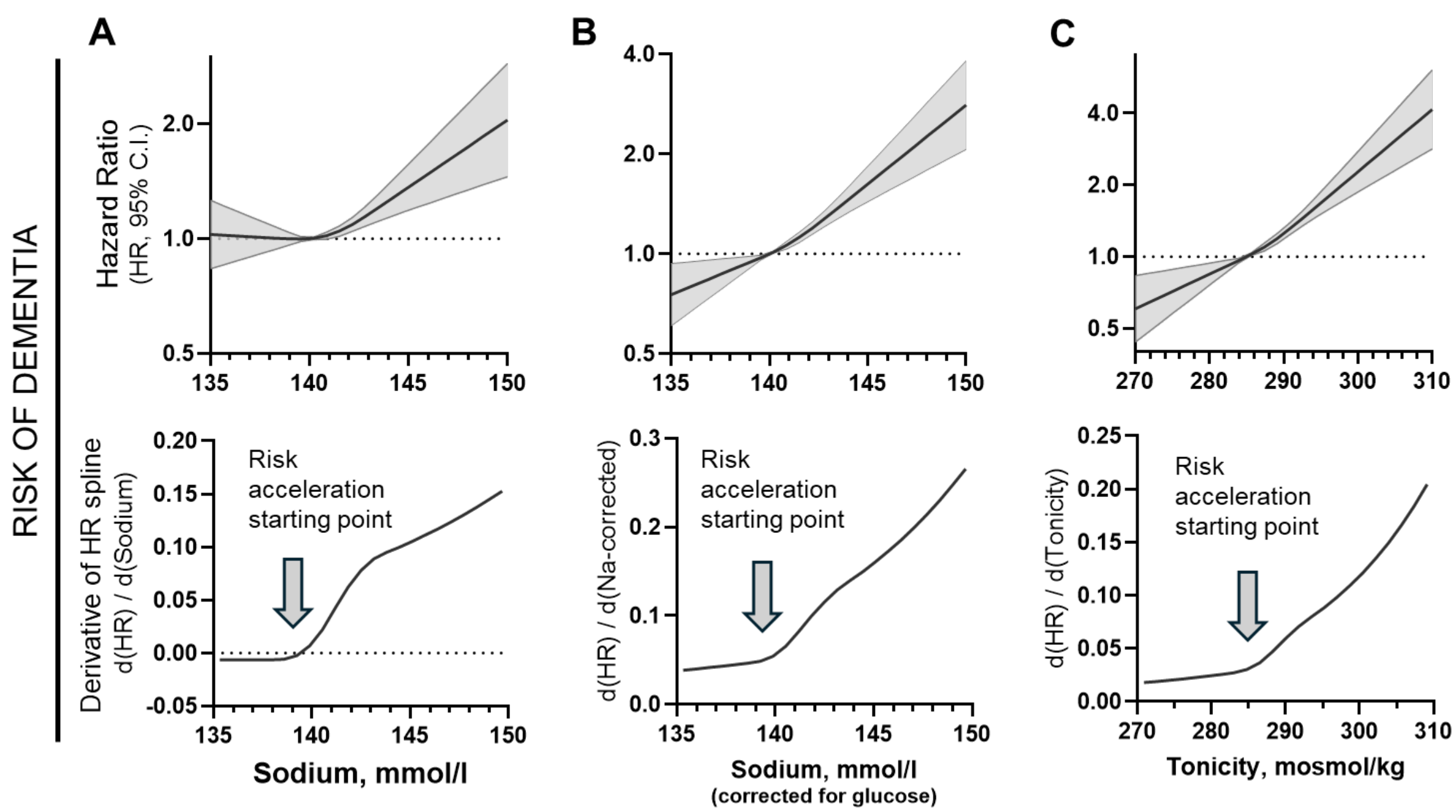

Figure S2 (related to Figure 2). Sensitivity analysis for the association between hydration-related biomarkers and dementia risk, excluding early dementia cases and short follow-up.
Hazard ratios (HRs) for incident dementia are shown as a function of hydration-related predictor variables modeled using restricted cubic splines from unadjusted Cox proportional hazards models. Panels A, B, and C correspond to: **A)** Serum sodium, **B)** Serum sodium corrected for glucose, and **C)** Serum tonicity. This analysis excludes 15 participants who developed dementia and 759 who were censored or lost to follow-up within 5 years of visit 2 to address potential reverse causation. Splines are plotted relative to 140 mmol/L for serum sodium and 285 mOsm/kg for serum tonicity.

The shaded areas represent 95% confidence intervals. P-values from likelihood ratio tests (LRTs), displayed on each panel, are derived from adjusted Cox proportional hazards models comparing models with and without the corresponding predictor variable. Covariates in the adjusted models include age, sex, education, ApoE4 status, smoking status, hypertension, LDL cholesterol, and BMI at visit 2. All three hydration-related variables significantly improved model fit, supporting the robustness of associations observed in the primary analysis. Notably, the shapes and inflection points of the HR splines closely resemble those shown in the main analysis (Figure 2), demonstrating the stability and consistency of the observed risk patterns.

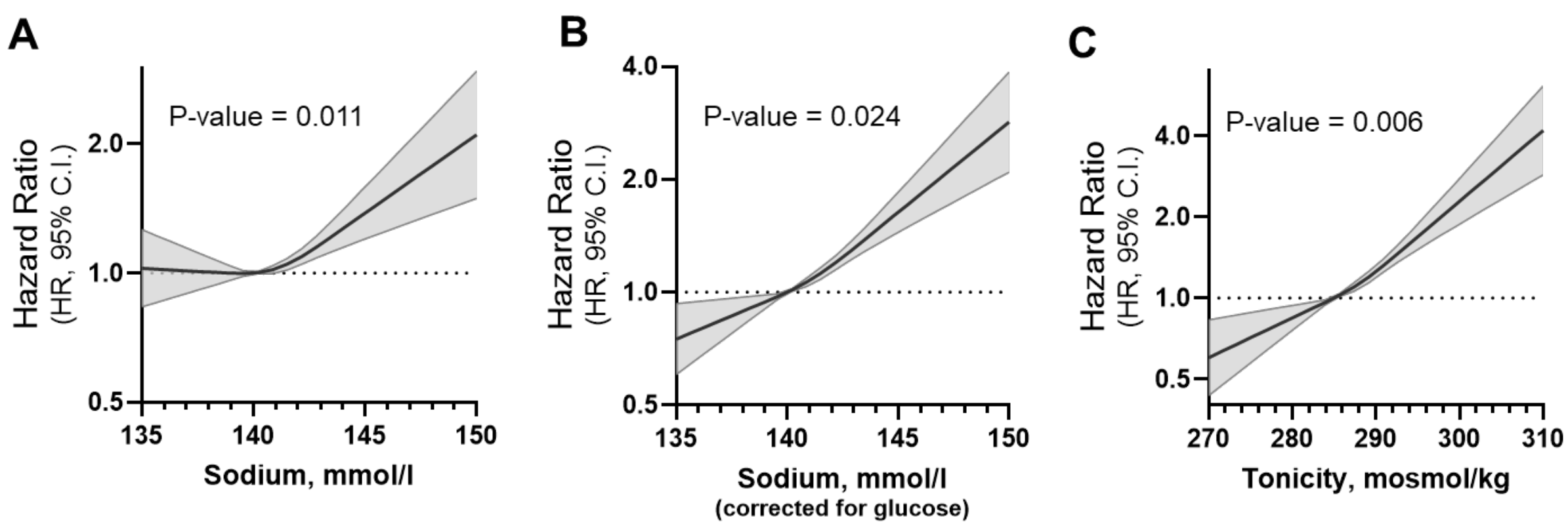

Figure S3. Modeling stroke's role in associations between hydration-related biomarkers and dementia risk. Conceptual diagram of the analyses shown on Figures 4, 5, S5.
(A) **Base model:** Cox proportional hazards (Cox PH) models with hydration biomarkers (serum sodium, glucose-corrected sodium, and serum tonicity) modeled using restricted cubic splines (RCS) to estimate associations with incident dementia. Model adjusted for age, sex, education, APOE4 genotype, smoking status, hypertension, LDL cholesterol, BMI, and race. Stroke is not included (Figure 2) .
(B) **Mediation test:** Stroke entered as a time-varying covariate (stroke(t)) to estimate stroke-independent associations (Figure 4).
(C) **Interaction test:** Effect modification by time-varying stroke assessed by adding a biomarker × stroke interaction (with RCS for the biomarker). Interaction was evaluated using likelihood-ratio tests (Figure S5) and visualized via HR splines stratified by stroke before dementia status (Figure 5).
All models are adjusted for age, sex, education, APOE4 genotype, smoking status, hypertension, LDL cholesterol, BMI, and race.

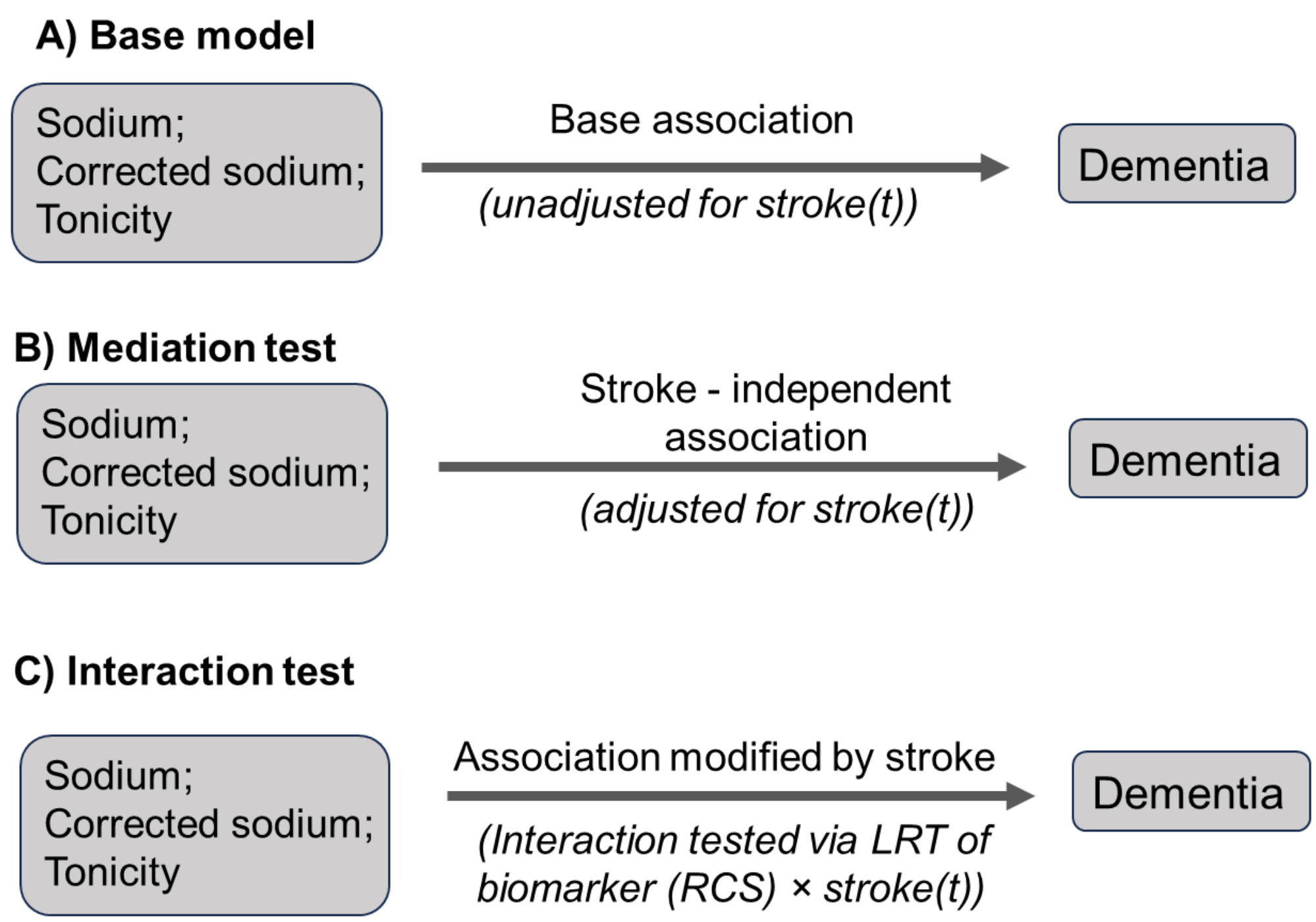

Figure S4. Stratified analysis of dementia hazard ratio (HR) splines by baseline diabetes status. The study cohort was divided into two groups based on diabetes diagnosis at visit 2: 11,872 individuals without diabetes **(A)** and 1,597 with diabetes **(B)**. HRs for incident dementia are shown as functions of hydration-related predictor variables modeled using restricted cubic splines from unadjusted Cox proportional hazards models. *Left panels*: serum sodium; *middle panels*: serum sodium corrected for glucose; r*ight panels*: serum tonicity. Splines are plotted relative to 140 mmol/L for serum sodium and 285 mOsm/kg for serum tonicity. The shaded areas represent 95% confidence intervals.

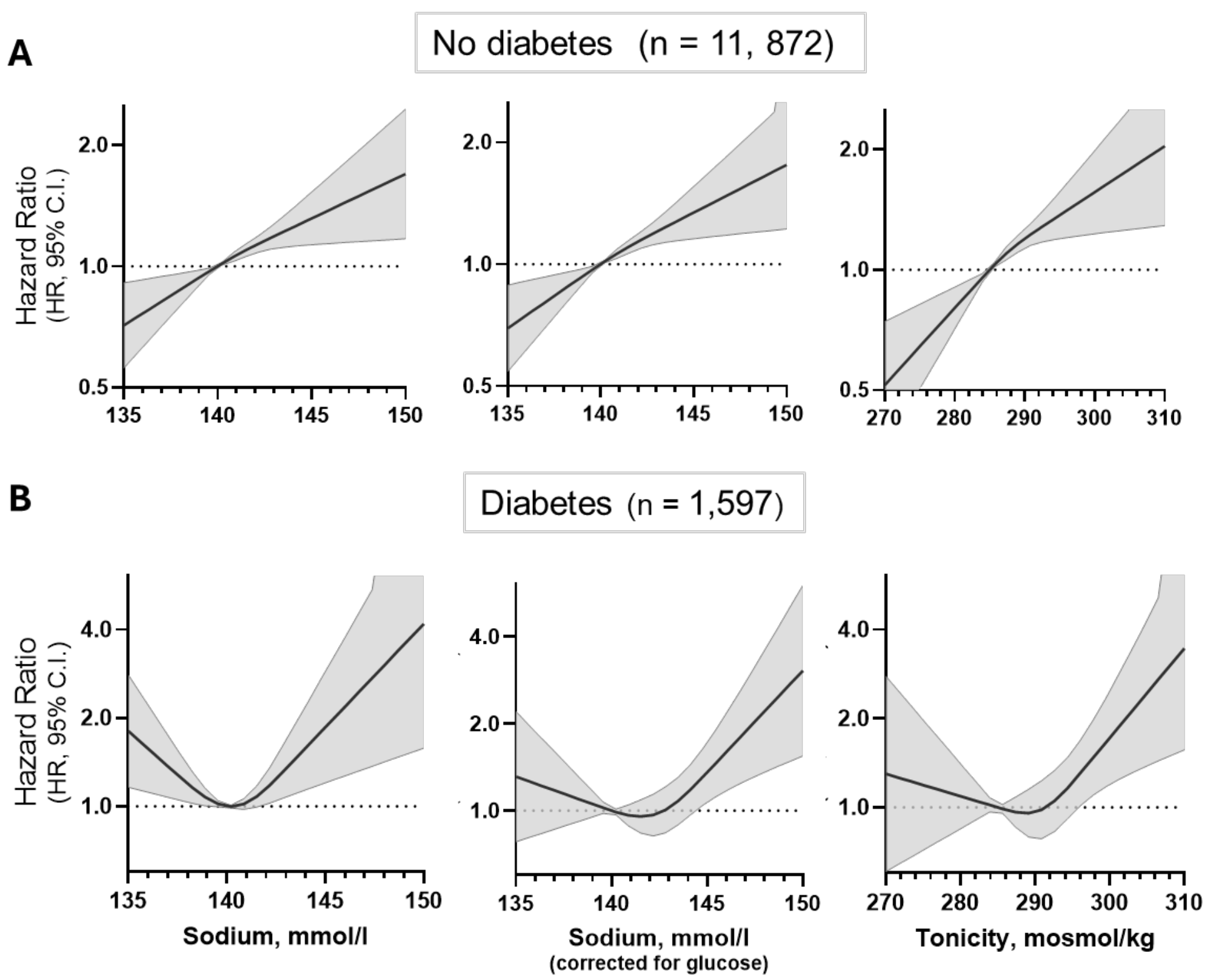

Figure S5. **Stratified analysis of dementia hazard ratio (HR) splines by baseline (A) hypertension status and (B) hypertension medication status.**

HRs for incident dementia are depicted as functions of hydration-related predictor variables, modeled using restricted cubic splines from unadjusted Cox proportional hazards models. The upper panels show serum sodium, the middle panels show serum sodium corrected for glucose, and the lower panels show serum tonicity. Splines are plotted relative to 140 mmol/L for serum sodium and 285 mOsm/kg for serum tonicity. The shaded areas represent 95% confidence intervals.

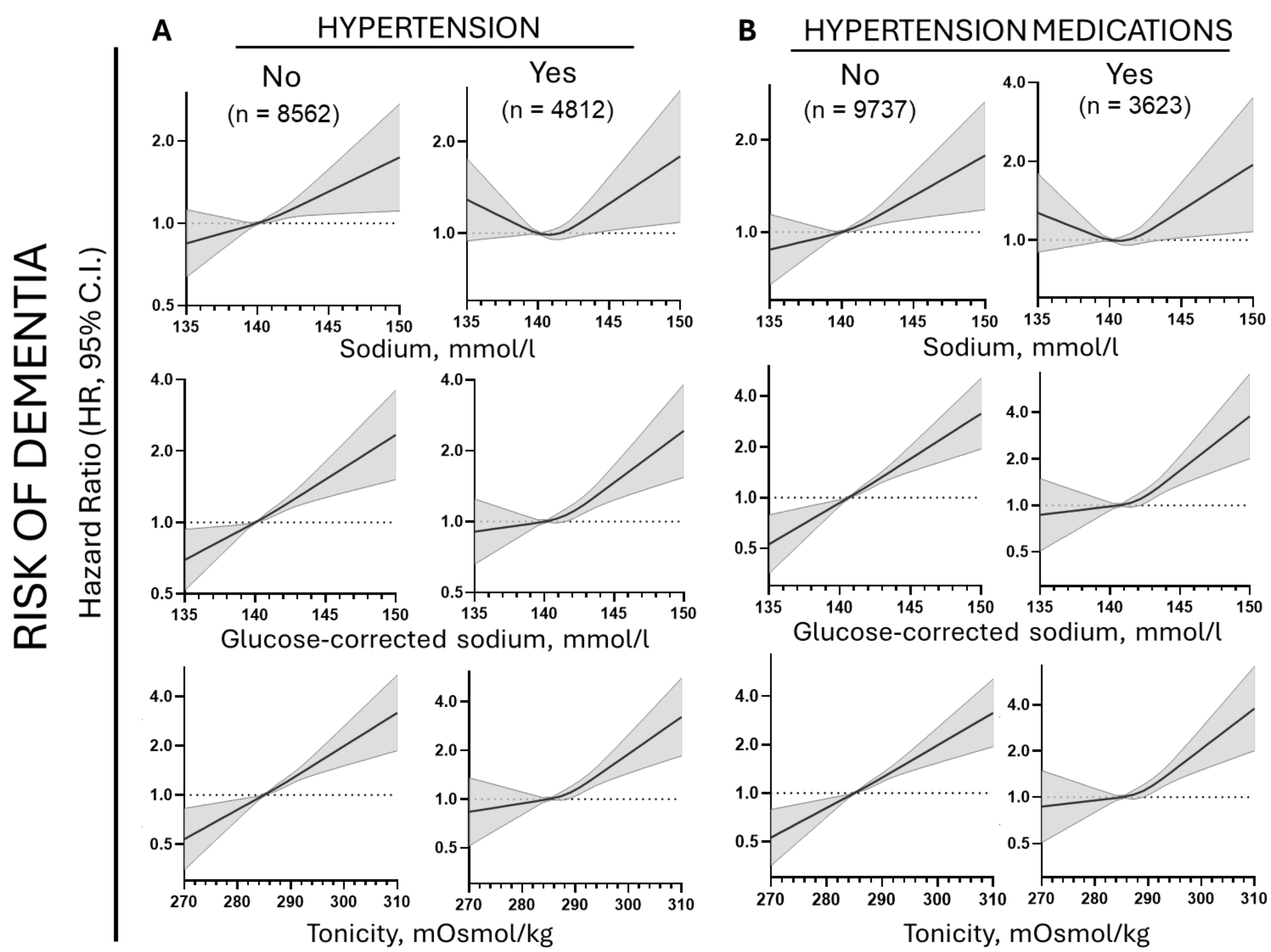

Figure S6. **Stratified analysis of dementia hazard ratio (HR) splines by baseline salt intake habits.** Salt intake habits were assessed at baseline using a survey question on how many shakes of salt participants typically add to meals at the table. HRs for incident dementia are presented as functions of hydration-related predictor variables, modeled using restricted cubic splines from Cox proportional hazards models. Splines are plotted relative to 140 mmol/L for serum sodium and 285 mOsm/kg for serum tonicity. Shaded areas represent 95% confidence intervals. *The trend for increased risk of dementia at higher levels of serum sodium and tonicity is observed in both salt intake strata.*

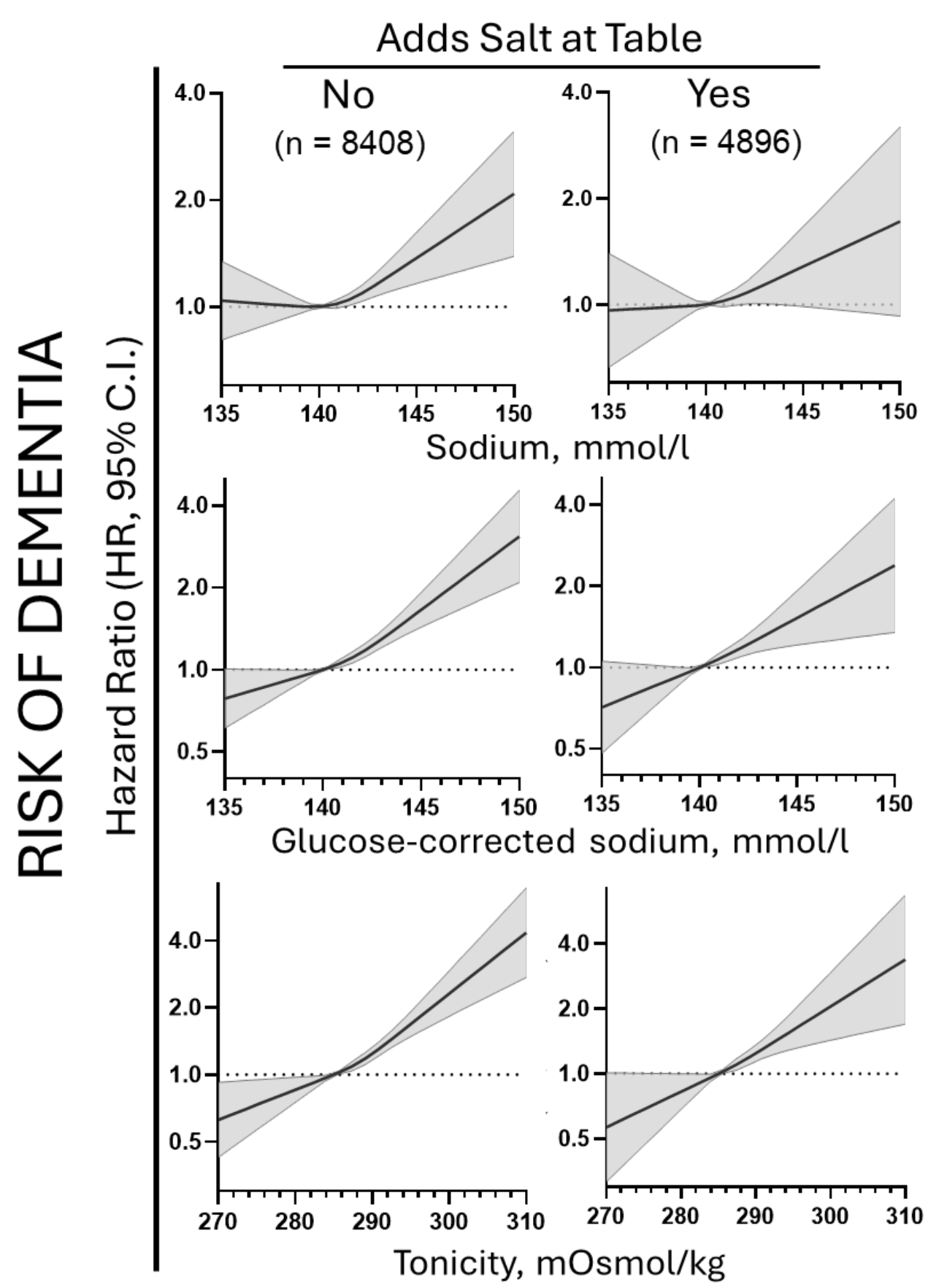

Supplemental Tables:

Table S1 (related to Methods section). Algorithm for calculating the glucose-corrected sodium exposure variable. Sodium corrected for hyperglycemia was derived from the average sodium and glucose concentrations at visits 1 and 2 using the Hillier method. The correction was performed as follows:

Corrected Sodium = Measured Sodium + 0.024 × (Glucose [mg/dL] – 100)

This corresponds to adding 1 mmol/L to the measured sodium concentration for every 42 mg/dL increase in glucose above 100 mg/dL.

		**Procedure for correction sodium for glucose**	
Glucose, mg/dl	Sodium to add, mmol/l: (calculated as 0.024 × (Glucose (mg/dl) – 100))	Glucose range, mg/dl	Sodium added, mmol/l
**100**	0	<142	+0
**142**	1.008	142-(<184)	+1
**184**	2.016	184-(<226)	+2
**226**	3.024	226-(<268)	+3
**268**	4.032	268-(<310)	+4
**310**	5.04	310-(<352)	+5
**352**	6.048	352-(<394)	+6
**394**	7.056	394-(<436)	+7
**436**	8.064	436-(<478)	+8
**478**	9.072	478-(<520)	+9
**520**	10.08	520-(<562)	+10
**562**	11.088	562-(<604)	+11
**604**	12.096	604-(<656)	+12
**646**	13.104	646-(<688)	+13

Table S2 (related to Table 1). Demographic and clinical characteristics of ARIC participants by serum glucose-corrected sodium group. Baseline characteristics at Visit 2 for participants included in the time-to-event analysis for dementia (N = 13,535). Participants were grouped by average serum glucose-corrected sodium concentration from Visits 1 and 2 into five equal-width intervals (135–150 mmol/L). Shown are demographic and clinical variables, including mean sodium concentrations at each visit, average glucose levels, and the dementia incidence rates observed during follow-up within each group. See Table S1 for algorithm use to calculate the glucose-corrected sodium exposure variable.

Baseline characteristics	All participants (N=13535)	Glucose-corrected Sodium groups, mmol/l (Average of V1&V2)				
		135-137.5 (N=488)	138-140.5 (N=5072)	141-143.5 (N=6724)	144-146.5 (N=1206)	147-150 (N=45)
Age, years – mean (SD)	57.1 (5.7)	55.8 (5.9)	56.5 (5.8)	57.4 (5.6)	58.1 (5.5)	58.4 (5.2)
Serum Na^+, mmol/l – mean (SD)						
Average of V1&2	140.9 (1.9)	137.0(0.6)	139.5(0.9)	141.8(1.1)	143.7(1.6)	145.1(3.2)
Visit 1	141.0 (2.4)	137.1(1.7)	139.5(1.5)	141.9(1.7)	143.9(2.3)	145.6(4.5)
Visit 2	140.8 (2.3)	136.8(1.7)	139.5(1.5)	141.6(1.7)	143.5(2.3)	144.7(3.8)
Glucose, mg/dL: Avg V1&2	111.0(38.1)	101 (11)	104 (23)	111 (37)	137 (68)	214 (124)
Female	7400 (55)	277 (57)	2562 (51)	3748 (56)	779 (65)	34 (76)
Male	6135 (45)	211 (43)	2510 (49)	2976 (44)	427 (35)	11 (24)
Race - no. (%)						
White	10301 (76)	421 (86)	4188 (83)	4986 (74)	693 (57)	13 (29)
Black	3234 (24)	67 (14)	884 (17)	1738 (26)	513 (43)	32 (71)
Current smoker – no. (%)						
Yes	3043 (23)	152 (31)	1178 (23)	1440 (21)	266 (22)	7 (16)
No	10457 (77)	335 (69)	3886 (77)	5268 (79)	931 (78)	37 (84)
Hypertension – no. (%)	4854 (36)	163 (33)	1629 (32)	2451 (37)	583 (48)	28 (62)
ApoE4 – no. (%)	4030 (31)	134 (29)	1459 (30)	2040 (32)	387 (33)	10 (23)
BMI† – mean (SD)	28.0 (5.4)	27.0 (5.0)	27.5 (5.1)	28.1 (5.5)	29.2 (5.7)	32.3 (7.9)
LDL Chol# – mean (SD)	134 (37)	125 (35)	131 (36)	135 (37)	142 (40)	141 (38)
Education - no. (%)						
< High School	2936 (22)	63 (13)	959 (19)	1534 (23)	365 (30)	15 (34)
HS or GED	5631 (42)	210 (43)	2096 (41)	2831 (42)	483 (40)	11(25)
>HS	4945 (37)	215 (44)	2012 (40)	2345 (35)	355 (30)	18 (41)
Diabetes-no. (%)	1597 (12)	21 (4)	337 (7)	849 (12)	366 (30)	24 (56)
Incident Dementia						
no. (%)	2965 (22.1)	76 (15.6)	1035 (20.6)	1546 (23.1)	298 (25.0)	10 (22.2)
Rate per 1000 person-years	10.4	7.3	9.5	10.9	13.1	13.2

* Hypertension is defined as systolic blood pressure >140 mmHg or diastolic blood pressure >90 mmHg, or use of antihypertensive medications; †BMI – body mass index, calculated as weight (kg) divided by height (m^2); # LDL Chol – low-density lipoprotein cholesterol, in mg/dL.

Table S3 (related to Table 1) . Demographic and clinical characteristics of ARIC participants by serum tonicity groups. Baseline characteristics at Visit 2 for participants included in the time-to-event analysis for dementia (N = 13,535). Participants were grouped by average serum tonicity from Visits 1 and 2 into five equal-width intervals (270-310 mosmol/kg). Shown are demographic and clinical variables, including mean sodium concentrations at each visit, average glucose levels, and the dementia incidence rates observed during follow-up within each group. Tonicity was calculated from serum sodium and glucose levels using the formula: Tonicity [mOsmol/kg]= 2 × Sodium[mmol/l] + Glucose [mg/dl]/18.

Baseline characteristics	All participants (N=13535)	Serum tonicity groups, mosmol/kg (Average of V1&V2)				
		[270- 278) (N=558)	[278-286) (N=5903)	[286-294) (N=6188)	[294-302) (N=845)	[302-310] (N=41)
**Age, years – mean (SD)**	57.1 (5.7)	55.7(5.9)	56.6(5.8)	57.5(5.6)	58.2(5.6)	58.6(5.0)
**Serum sodium, mmol/l – mean (SD)**						
**Average of V1&2**	140.9 (1.9)	137.1(0.7)	139.8(0.9)	142.0(1.2)	143.6(2.1)	144.0(3.9)
**Visit 1**	141.0 (2.4)	137.2(1.6)	139.7(1.6)	142.1(1.8)	143.9(2.7)	144.2(5.5)
**Visit 2**	140.8 (2.3)	137.0(1.7)	139.8(1.6)	141.9(1.8)	143.3(2.7)	143.8(4.1)
**Glucose, mg/dL: Avg V1&2**	111.0(38.1)	99 (11)	103 (19)	113 (38)	155 (78)	260 (139)
**Female**	7400 (55)	332(59)	3045(52)	3445(56)	548(65)	30(73)
**Male**	6135 (45)	226(41)	2858(48)	2743(44)	297(35)	11(27)
**Race - no. (%)**						
**White**	10301 (76)	485(87)	4853(82)	4516(73)	435(51)	12(29)
**Black**	3234 (24)	73(13)	1050(18)	1672(27)	410(49)	29(71)
**Current smoker – no. (%)**						
**Yes**	3043 (23)	164(29)	1383(23)	1310(21)	182(22)	3(10)
**No**	10457 (77)	393(71)	4509(77)	4765(79)	654(78)	36(90)
**High BP – no. (%)**	4854 (36)	169(30)	1851(31)	2350(38)	454(54)	30(73)
**ApoE4 – no. (%)**	4030 (31)	152 (29)	1718 (30)	1878 (32)	270 (33)	12 (31)
**BMI† – mean (SD)**	28.0 (5.4)	26.7 (5.1)	27.4 (5.0)	28.3 (5.5)	30.0 (5.9)	32.9 (7.6)
**LDL Chol# – mean (SD)**	134 (37)	125 (35)	131 (36)	136 (37)	143 (41)	147 (44)
**Education - no. (%)**						
**< High School**	2936 (22)	75(13)	1104(19)	1458(24)	285(34)	14(35)
**HS or GED**	5631 (42)	238(43)	2449(42)	2614(42)	320(38)	10(25)
**>HS**	4945 (37)	245(44)	2342(40)	2104(34)	238(28)	16(40)
**Diabetes-no. (%)**	1597 (12)	19 (3)	349 (6)	861 (14)	341 (41)	27 (69)
**Incident Dementia**						
**no. (%)**	2965 (22.1)	91 (16.4)	1221(20.9)	1441(23.5)	204(24.6)	8(19.5)
**Rate per 1000 person-years**	10.4	7.7	9.5	11.1	13.5	12.4

* High BP is defined as systolic blood pressure >140 mmHg or diastolic blood pressure >90 mmHg, or use of antihypertensive medications; †BMI – body mass index, calculated as weight (kg) divided by height (m^2); # LDL Chol – low-density lipoprotein cholesterol, in mg/dL.

Table S4. Assessment of salt intake in relation to serum sodium and tonicity among ARIC participants based on the habit of adding salt at the table to already prepared food.

Salt intake habits were assessed at baseline using a survey question regarding how many shakes of salt participants typically add to a meal at the table. Participants were grouped by average serum sodium concentration, glucose-corrected sodium, and tonicity from Visits 1 and 2 into five equal-width intervals. The percentage of participants who reported adding salt at the table is presented for each group. *A higher proportion of ARIC participants in the lower serum sodium and tonicity groups reported adding salt at the table to already prepared food suggesting that salt intake is not main driver of higher serum sodium in ARIC study participants..*

% of participants adding salt at the table to already prepared meal					
**All participants**	**Serum sodium groups, mmol/l (Average of V1&V2)**				
	135-137.5	138-140.5	141-143.5	144-146.5	147-150
**37%**	38%	40%	35%	30%	26%
	**Glucose-corrected Serum sodium groups, mmol/l**				
	40%	41%	35%	28%	24%
	**Serum tonicity groups, mosmol/kg**				
	[270-278)	[278-286)	[286-294)	[294-302)	[302-310)
	41%	40%	35%	37%	25%

Table S5. Assessment of baseline kidney function in ARIC participants based on eGFR measured at Visit 2.

This analysis was performed on the cohort used for sensitivity analysis for reverse causation, excluding dementia cases within 5 years after Visit 2 baseline (N = 12,761). Participants were grouped by average serum sodium concentration, glucose-corrected sodium, and tonicity from Visits 1 and 2 into five equal-width intervals. There were no major differences in mean eGFR between groups. A total of 233 participants (1.8%) had baseline eGFR < 60, with a higher prevalence observed in the two highest serum sodium and tonicity groups.

Conclusion: Inefficient renal sodium excretion in individuals with chronic kidney disease may contribute to the observed associations between higher serum sodium/tonicity and increased dementia risk in these groups. Sensitivity analysis excluding these 233 individuals is warranted; see Table 2 for results.

eGFR (mean (SD) and proportion of participants with CKD					
**eGFR**	**Serum sodium groups, mmol/l (Average of V1&V2)**				
	135-137.5	138-140.5	141-143.5	144-146.5	147-150
mean(SD)	98(17):	96(15)	96(15)	96(17)	101(14)
% < 60 (n)	2.2% (14)	1.8% (94)	1.6% (99)	3.3% (26)	0% (0)
	**Glucose-corrected Serum sodium groups, mmol/l**				
mean(SD)	98(16)	96(15)	96(15)	96(18)	95(20)
% < 60 (n)	2.0% (9)	1.6% (79)	1.6% (102)	3.7% (40)	7.5% (3)
	**Serum tonicity groups, mosmol/kg**				
	[270-278)	[278-286)	[286-294)	[294-302)	[302-310)
mean(SD)	97(15)	96(15)	96(16)	97(19)	94(20)
% < 60 (n)	2.1% (11)	1.6% (88)	1.8% (104)	3.6% (27)	8.5% (3)

Table S6. Sensitivity analysis of model improvement after additional covariate adjustment.

The table presents P-values from likelihood ratio tests (LRTs) comparing model fit with and without each exposure variable. Models were additionally adjusted for potential confounders, including salt intake, alcohol intake, physical activity, and kidney function, assessed either by adjustment for eGFR as a covariate or by excluding individuals with eGFR <60 mL/min/1.73 m². This analyses tests whether serum sodium, glucose-corrected sodium, and serum tonicity remain significant predictors of dementia after adjustment for all other covariates in the Cox models. *Across models, all three exposure variables remained significant predictors of dementia risk, with tonicity showing strongest association.*

*P-values for model improvement when evaluating hydration-related biomarkers (serum sodium, glucose-corrected sodium, and tonicity) as predictors of dementia risk in ARIC participants.**			
	**Serum sodium**	**Corrected Sodium**	**Tonicity**
**Cohort 1 (n=12,761): sensitivity analysis cohort**			
**a)** Base model #	0.011	0.024	0.006
**b)** Base model + additional adjustment for salt intake, eGFR, alcohol intake and physical activity	0.013	0.016	0.003
**Cohort 2: Excluding 233 participants with eGFR < 60 from Cohort 1**			
Model (b) from Cohort 1 above	0.010	0.026	0.006

*P-values are from likelihood ratio tests (LRTs) comparing model fit with and without the corresponding exposure variable.

Base model includes adjustment for age, sex, education, ApoE4 genotype, smoking status, hypertension, LDL cholesterol, and BMI measured at Visit 2. Cohort 1 excluded 15 dementia cases and 759 participants who were lost to follow-up or died within 5 years of Visit 2.

Table S7 (related to Figure 6). Interaction analysis between hydration-related biomarkers and demographic/clinical variables. This table reports likelihood-ratio test (LRT) p-values from Cox proportional hazards models of incident dementia, comparing models with vs. without interaction terms between each hydration biomarker - serum sodium, glucose-corrected sodium, and serum tonicity (each modeled with restricted cubic splines, 3 knots) - and selected modifiers. **Stroke** was modeled as a **time-varying covariate**; interactions were specified as biomarker x stroke.

**Interaction →**	**Sodium**	**Sodium, corrected**	**Tonicity**
↓	*p values for interaction*		
**Time-varying Stroke**	**0.02**	0.06	0.09
**Race**	0.44	0.61	0.42
**Sex**	0.83	0.58	0.64
**ApoE4**	0.89	0.39	0.53
**Hypertension**	0.61	0.96	0.9
**Diabetes**	0.06	0.29	0.2
**eGFR**	0.36	0.8	0.87

Table S8 (related to Figure 7A). Calculated serum tonicity values corresponding to different combinations of sodium and glucose concentrations, used for dementia risk estimation in conjunction with Table S7. These values were applied to derive predicted dementia risk ranges for the sodium–glucose subgroups shown in Figure 6A.

		Glucose, mg/dl							
		**80**	**100**	**126**	**140**	**180**	**220**	**260**	**300**
Sodium, mmol/l	**135**	274	276	277	278	280	282	284	287
	**136**	276	278	279	280	282	284	286	289
	**137**	278	280	281	282	284	286	288	291
	**138**	280	282	283	284	286	288	290	293
	**139**	282	284	285	286	288	290	292	295
	**140**	284	286	287	288	290	292	294	297
	**141**	286	288	289	290	292	294	296	299
	**142**	288	290	291	292	294	296	298	301
	**143**	290	292	293	294	296	298	300	303
	**144**	292	294	295	296	298	300	302	305
	**145**	294	296	297	298	300	302	304	307
	**146**	296	298	299	300	302	304	306	309
	**147**	298	300	301	302	304	306	308	311
	**148**	300	302	303	304	306	308	310	313
	**149**	302	304	305	306	308	310	312	315
	**150**	304	306	307	308	310	312	314	317
		Tonicity = 2 x Sodium + Glucose/18							

Table S9 (related to Fig. 7A). Hazard ratios for incident dementia across serum tonicity values relative to the 270–286 mosmol/kg reference range

Hazard ratios (HRs) and 95% confidence intervals (CIs) for incident dementia were estimated using a Cox proportional hazards model with serum tonicity modeled as a restricted cubic spline (3 knots), adjusted for age at Visit 2, with age centered at the cohort median. HRs for tonicity values between 286 and 310 mosmol/kg are expressed relative to the reference interval of 270–286 mosmol/kg, which is shaded in the table.

The reference category was defined by averaging the log hazard ratios across the 270–286 range, and all spline-based predictions were centered on this mean value (279 mosmol/kg). Log hazard ratios were then exponentiated to derive HRs and corresponding 95% CIs. These values were used to generate the predicted dementia risks based on serum tonicity for the sodium-glucose groups shown in Figure 7A.

Tonicity, mosmol/kg	HR	C.I. 95%, lower end	C.I. 95%, upper end
270	0.96	0.69	1.33
271	0.96	0.71	1.31
272	0.97	0.73	1.29
273	0.97	0.75	1.27
274	0.98	0.77	1.25
275	0.98	0.79	1.23
276	0.99	0.81	1.21
277	0.99	0.83	1.19
278	1.00	0.85	1.17
279	1.00	0.87	1.16
280	1.01	0.90	1.14
281	1.02	0.92	1.12
282	1.02	0.95	1.10
283	1.03	0.97	1.09
284	1.03	0.99	1.08
285	1.04	1.01	1.07
286	1.05	1.03	1.08
287	1.07	1.04	1.10
288	1.10	1.06	1.13
289	1.13	1.10	1.17
290	1.18	1.14	1.21
291	1.23	1.20	1.27
292	1.29	1.25	1.34
293	1.36	1.30	1.43
294	1.43	1.34	1.53
295	1.51	1.39	1.64

296	1.59	1.44	1.76
297	1.68	1.49	1.89
298	1.76	1.54	2.02
299	1.86	1.59	2.17
300	1.96	1.64	2.33
301	2.06	1.70	2.50
302	2.17	1.75	2.68
303	2.28	1.81	2.88
304	2.40	1.87	3.09
305	2.53	1.93	3.31
306	2.67	2.00	3.56
307	2.81	2.06	3.82
308	2.96	2.13	4.09
309	3.11	2.20	4.39
310	3.28	2.28	4.72
